# FDNet: A Lightweight Feature-Dominated U-Net for Hippocampus Segmentation in Brain MRI

**DOI:** 10.3390/diagnostics16213563

**Published:** 2026-10-29

**Authors:** Mehmet Halis Korkmaz, Özkan Bingöl, the Alzheimer’s Disease Neuroimaging Initiative

**Affiliations:** 1 Department of Mathematics, Institute of Science, Gümüşhane University, 29010 Gümüşhane, Türkiye; 2 Department of Software Development, Faculty of Science, Karadeniz Technical University, 61080 Trabzon, Türkiye; bingolo@ktu.edu.tr

**Keywords:** hippocampus segmentation, deep learning, U-Net, feature domination, magnetic resonance imaging, Alzheimer’s disease, brain MRI

## Abstract

**Background/Objectives:** Hippocampus segmentation in brain MRI is challenging because the structure is small, irregularly shaped, and poorly contrasted against surrounding tissue. Increasingly complex U-Net variants improve accuracy at computational cost. We investigated whether modifying the relative contribution of encoder features within skip connections can improve performance without additional attention or gating modules. **Methods:** We propose FDNet, a lightweight feature-dominated U-Net that concatenates multiple identical copies of encoder feature maps with the corresponding decoder features. Three variants (FDNetX2, FDNetX3, and FDNetX4) were evaluated on sagittal MRI slices from ADNI, the Medical Segmentation Decathlon, and IBSR using five-fold cross-validation, assessed with overlap metrics and per-slice false-positive/false-negative counts. To address potential subject-level leakage in the slice-level evaluation, a subject-grouped assessment with a fixed held-out test set was additionally conducted on ADNI, with re-implemented baselines and nnU-Net v2. **Results:** Under the slice-level protocol, FDNet achieved Dice coefficients of 0.9339 on ADNI, 0.9470 on Decathlon, and 0.9477 on IBSR, outperforming re-implemented U-Net, Attention U-Net, and SegNet baselines with substantially fewer parameters. Under the subject-grouped protocol, the best FDNet variant reached a held-out test Dice of 0.9050 versus 0.9019 for nnU-Net. All variants significantly outperformed the three baselines after Holm correction (adjusted *p* < 0.0001); their advantage over nnU-Net was not significant. **Conclusions:** Repeated encoder features within skip connections can provide competitive hippocampus segmentation with a relatively small parameter budget. Because all results come from curated research datasets without external or cross-dataset validation, they indicate competitive performance under the evaluated conditions rather than clinical readiness.

## 1. Introduction

The hippocampus is a structure of the medial temporal lobe [1] that is critical for long-term memory and contributes to the spatial aspects of short-term memory [2]. It is affected in schizophrenia [3], Alzheimer’s disease [4,5,6], epilepsy [5,7], and related disorders, and hippocampal atrophy is an early clinical finding in these conditions [8,9,10]. Therefore, an accurate measurement of hippocampal shape and volume is central to the investigation and monitoring of these diseases.

Atrophy is assessed volumetrically on anatomical MRI, and when performed manually, it requires a time-consuming delineation of the structure on each slice of every scan [9]. This task is difficult because the hippocampus is small, no sharp intensity boundary separates it from adjacent tissue, and its shape is irregular and varies substantially between individuals [11]. Therefore, computer-aided approaches have been pursued for decades to overcome these challenges. Early methods drew on classical computer graphics and computer vision [12,13,14] and did not achieve clinically useful accuracy; for example, the elastic model-based segmentation of Kelemen et al. [14] reported a 57% overlap.

Machine learning has improved the accuracy of hippocampal segmentation. Label-fusion and atlas-based methods [15] reached a Dice similarity coefficient (DSC) of 0.887 [16] and 0.881 [17] on ADNI, and classification-based methods reached 0.831 [18]. With the advent of convolutional neural networks (CNNs), CNN- and U-Net-based methods have become dominant [15], reporting DSC values of 0.9098 [19] and 0.87 [20] on the same dataset.

U-Net [21] comprises a contracting encoder and expanding decoder. The encoder extracts increasingly abstract features through convolution and pooling. The decoder restores the resolution through transposed convolution while concatenating, at each level, the feature maps carried across from the corresponding encoder level by a skip connection. Therefore, the segmentation mask is produced from fine-grained details and coarse spatial context. The success of this design has motivated the development of many variants, including UNet++, which adds nested dense skip connections [22], Attention U-Net, which inserts attention gates between the encoder and decoder [23], and SegNet, which places convolutional layers on both sides of the encoder–decoder pair [24]. These variants can improve accuracy; however, every additional module or connection increases the computation, and the cost increases with the dataset size.

Studies published since 2024 have pushed this trade-off in two directions. On one hand, capacity is continually being added: state-space (Mamba) formulations have been combined with diffusion priors for volumetric segmentation [25], and transformer–capsule hybrids have been proposed to model long-range structures that are missed by convolutions [26]. On the other hand, an explicit efficiency literature has emerged, motivated by the observation that many high-capacity segmentation networks cannot be deployed where they are needed: neural cellular automata reach competitive accuracy with a small fraction of the parameters of a U-Net [27], and for the hippocampus, Xiao et al. [28] combined multi-scale convolutional attention with a lightweight vision transformer and reported better accuracy than heavier alternatives with fewer parameters. The present study follows an efficiency-oriented direction but investigates a different mechanism: modifying existing skip connections without introducing additional learnable modules while maintaining a substantially smaller overall parameter budget than that of larger baseline architectures.

We followed a different approach. Instead of adding modules, we changed the balance of information that already crosses the skip connection. In the standard design, each encoder feature map is concatenated once with the upsampled decoder feature map so that fine detail is combined with spatial context in a ratio of one to two. We concatenated several identical copies of the encoder feature map instead, which raised the relative weight of encoder detail in the subsequent convolutions. We call the resulting network the Feature-Dominated U-Net (FDNet). Because the mechanism adds no learnable modules, FDNet can be built with fewer layers than the networks it is compared against and is therefore substantially smaller.

The specific contributions of this work are as follows:

- We introduce feature domination, a skip-connection modification that increases the influence of encoder details on the decoder by concatenating repeated copies of the encoder feature map, and we formalize it as a single operator with an adjustable degree of domination.
- We show that the mechanism does not increase the network’s representational capacity but instead scales the encoder branch at initialization and during optimization, which accounts for the intermediate number of copies being the best-performing setting, and we quantify its parameter cost.
- We motivate and evaluate the method through the balance between false-negative and false-positive predictions rather than through overlap alone and report mean per-slice false-negative and false-positive pixel counts (FNPr and FPPr) to explicitly characterize this balance.
- We evaluate three degrees of domination on three public datasets (ADNI, Medical Segmentation Decathlon, and IBSR) under a common five-fold protocol, alongside U-Net, Attention U-Net, SegNet, and a published attention-based method.
- We show that the resulting networks are one to two orders of magnitude smaller than the baseline networks, while achieving a higher DSC than all of them on all three datasets.

The remainder of this paper is organized as follows. The Related Work Section reviews the relevant literature. Section 2 describes the datasets, proposed architecture, baseline methods, training configuration, and evaluation metrics used in this study. Section 3 presents the quantitative and qualitative results, including subject-group evaluations and computational efficiency analyses. Section 4 discusses the results. Section 5 outlines the study limitations. Finally, Section 6 concludes the paper.

### Related Work

Automatic hippocampal segmentation has been attempted using a wide range of methods, and the highest reported accuracies have been obtained using U-Net-based designs. Because the proposed method is also U-Net-based, the review below concentrates on this family while also covering other methods evaluated on the three datasets used in this study; therefore, the review focuses on methods that are most directly relevant to the comparisons reported in Section 3.

U-Net-based networks exist in both 2D and 3D. Most published studies segment 2D images. Although MRI is intrinsically volumetric and slice-wise processing discards variations along the through-plane axis, 2D segmentation is not necessarily less accurate [29], and volumetric processing has a much higher computational cost and parameter count [30]. Following the majority of the literature and to maintain a direct comparison with published results, the datasets in this study were prepared as 2D sagittal slices.

Chen et al. (2017) proposed a two-part architecture, U-Seg-Net, followed by Ensemble-Net [30]. They sliced each volume along nine orientations (sagittal, coronal, axial, and six diagonal views), segmented each set separately, and combined the outputs, achieving a DSC of 0.8947 on 110 healthy ADNI scans. Cao et al. (2018) proposed a multitask network that predicts the Mini-Mental State Examination score alongside segmentation [31]. Patches of 72 × 72 × 72 voxels were extracted from linearly registered volumes to limit class imbalance, and right hippocampi were reflected to match the left-hand orientation. Five-fold cross-validation of 797 ADNI scans yielded a DSC of 0.893.

Hou et al. (2019) proposed a 3D U-Net-based network built from an interaction block, which provides two interaction pathways between the encoder and decoder and a sampling block combining a feature-extracting convolution with a fully connected path [32]. On 500 ADNI scans, DSC was 0.919. Folle et al. (2019) investigated 3D U-Net variants for semantic segmentation using dilated convolutions to widen the receptive field and combine decoder outputs to provide deep supervision [33]. Five-fold cross-validation of 263 Decathlon scans yielded a DSC value of 0.882.

Brusini et al. (2020) proposed a combined 2D U-Net pipeline in which the statistical shape of the hippocampus entered the network as contextual information, and three orientation-specific segmentations were averaged into a volumetric estimate, which was used to crop the center of mass [34]. The first step was repeated on the cropped volume, and the cropped volume and shape estimates were combined for the final prediction. For 5948 ADNI images, the DSCs were 0.8506 (left) and 0.8606 (right). The study in [35] combined nnU-Net [36] with transformer mechanisms by inserting a Vision Transformer between the encoder and decoder blocks and feeding it skip-connection outputs. After training on 260 Decathlon scans using k-fold cross-validation, the 2D model achieved DSC of 0.8976.

Yousefi-Banaem and Malekzadeh (2021) modified the layer configuration and hyperparameters of U-Net [37]. Working in the coronal plane on 6336 images from 100 of 135 ADNI scans, they reported a DSC of 0.9230. Huang et al. (2023) identified two weaknesses of existing U-Net variants for this task: the shallow manner in which low- and high-level features are merged and the insufficient suppression of the background structure [38]. Their multilevel feedback chain network was evaluated using k-fold cross-validation on 2D slices from all three datasets used here, reaching a DSC of 0.9311 (Decathlon, volumes rescaled to 32 × 32 × 32 with 12–20 slices each), 0.9236 (ADNI, 135 subjects), and 0.9193 (IBSR). Because this study used the same three datasets and a comparable slice-based protocol, it is the closest point of comparison for the results reported below. SegNet [24] was also used as a comparison method for this task. Cai et al. (2021) reported a DSC of 0.834 for their method and 0.707 for SegNet on NITRC data, and Zhu et al. (2022) reported 0.836 and 0.722, respectively [39,40].

Several methods have been evaluated on these datasets, beyond the U-Net family of models. On the IBSR, Rousseau et al. (2011) proposed a patch-based label-propagation method, achieving a DSC of 0.83 [41], and Zarpalas et al. (2014) reported a hybrid active contour model on a multi-atlas basis, achieving 0.852 [42]. On ADNI, Thyreau et al. (2018) reported 0.85 with a deep CNN trained by transferring algorithmic knowledge [43]; Liu et al. (2020) reported 0.87 with a 3D multi-model dense network [44]; and Shi et al. (2021) reported 0.9124 on ADNI and 0.9038 on Decathlon with a discriminative feature network built on a hierarchical attention mechanism [11], in which high- and low-level features were extracted separately in the encoder and recombined using a cross-connected attention module in the decoder. Yue et al. (2025) fine-tuned a hybrid spiking neural network with a surrogate-gradient procedure on 24 × 56 × 48 patches from 110 ADNI scans, reaching 0.7809 in 2D and 0.8186 in 3D [45]. Ataloglou et al. (2019) combined three orthogonal slicing orientations with transfer learning and coupled Deform and Refine networks, achieving 0.9015 on 100 ADNI scans and 0.8835 on 35 MICCAI scans [46].

Overall, this body of work responds to the limitations of the plain U-Net on the hippocampus in one consistent way: by adding machinery. Attention gates were inserted between the encoder and decoder [23]; attention was then made multi-scale and applied at several resolutions at once [47]; squeeze-and-excitation blocks were added to recalibrate channels in volumetric brain segmentation [48]; a hybrid attention mechanism with cross-dimensional interaction was built for the hippocampus specifically [49]; an adversarial discriminator was added under dual multilevel constraints for hippocampal sclerosis, where the anatomy departs furthest from the healthy template [50]; and fuzzy-logic post-processing was combined with a regularized U-Net so that segmentation could feed directly into subtype classification [51]. Even work that targets the skip connection itself follows the same pattern: Wang et al. (2024) attribute the residual error of U-Net to a semantic gap between the encoder and decoder features being concatenated and close it by making the skip connection learnable, that is, by inserting a trainable transform into the pathway [52]. Sohail and Anwar (2022) reached the same diagnosis from the opposite direction, reporting that a modified U-Net still yields irregular boundaries on the hippocampus [53]. Our reading of this literature is that the diagnosis is correct and the prescription is not the only one available: if the encoder features arriving at the decoder are underweighted relative to the upsampled context, the imbalance can be corrected by changing how many times those features are presented, which requires no trainable transform, no gate, and no discriminator. That is the possibility this paper tests, and it is what distinguishes it from the learnable-skip-connection line of work to which it is closest.

Studies published since 2024 have been divided sharply according to whether they segment 2D slices or whole 3D volumes, and this distinction matters because a volume-wise DSC and a slice-wise DSC are not the same quantity: the volumetric score is dominated by the large mid-body cross-sections, whereas a slice-wise average weights the thin head and tail slices equally. Among the volumetric methods, Xiao et al. (2024) proposed Light3DHS [28], which places a multiscale convolutional attention module in the encoder and a lightweight vision transformer above it, and reported more accurate hippocampus segmentation than competing methods on three hippocampal datasets with fewer parameters. Khan et al. (2025) combined contrast-limited adaptive histogram equalization with a wavelet-based enhancement in a resource-efficient 3D U-Net and reported a DSC of 0.8838 on the EADC-ADNI HarP set [54]. The same group showed that the choice of preprocessing pipeline alone moves the DSC by more than a point on HarP and changes the ranking of methods under transfer from Decathlon, with boundary distance and overlap responding to preprocessing in opposite directions [55]. Zhu et al. (2024) addressed the finer-grained problem of hippocampal subfields with a dual-branch network [56], and Liu et al. (2024) combined an atlas prior with a lattice Boltzmann model for subregion segmentation, reporting a median DSC by subregion rather than for the structure [57]. Gao et al. (2024) took a different direction again, using geodesic shape regression to impose temporal consistency on longitudinal hippocampal segmentation [58]. None of these results is directly comparable with the slice-wise scores reported in Section 3; therefore, they are discussed here but excluded from the tabulated comparisons. 

Two recent studies evaluated 2D slices, and are therefore comparable to this study. Wei et al. (2025) proposed CSDA-UNet [59], which cascades a spatial attention module acting on the deepest encoder features with an inter-slice attention module that propagates information between neighboring slices, recovering some of the volumetric consistency that slice-wise processing loses. Trained on 18,900 axial slices from 100 ADNI subjects with a subject-level split and evaluated on 6615 slices from the remaining 35, it reported DSC 0.9512 on ADNI—higher than any result reported here—and 0.8963 on the Decathlon validation split. Their pipeline differs from ours in four respects that bear on the comparison: the slices are axial rather than sagittal; all 189 slices per subject are used, although only approximately 30 contain hippocampal tissue; intensities are equalized with contrast-limited adaptive histogram equalization and denoised with a Gaussian filter before training; and the network carries 7.76 M parameters, 15 times the largest FDNet variant. The effect of the third is quantified in their own ablation: with the enhancement removed, their U-Net falls from DSC 0.9365 to 0.7188 and CSDA-UNet from 0.9512 to 0.8820; thus, intensity preprocessing accounts for a larger share of their reported accuracy than the attention modules. Pusparani et al. (2026) compared U-Net, YOLO-v8, and DeepLab-v3 on 7859 ADNI slices from 300 subjects, splitting 80:10:10 at the subject level, and reported results stratified by diagnosis: U-Net achieved the best Dice in every stratum (0.784 for Alzheimer’s disease, 0.890 for mild cognitive impairment, and 0.858 for normal cognition), whereas YOLO-v8 was markedly worse than U-Net [60]. Their stratification is informative for the present study in a way that their absolute values are not, since it shows that segmentation difficulty tracks disease stage—atrophic hippocampi are harder—which is a source of variance that pooled averages such as ours conceal. A third study, PCFR-Net [61], reported a DSC of 0.9386 on ADNI and 0.9274 on Decathlon with a parallel cascaded feature-reconstruction network combining self-attention with convolution; however, the openly available record did not state whether the reported scores were slice-wise or volumetric.

Several additional 2D slice-based hippocampus segmentation studies published between 2022 and 2026 directly bear on the evaluation protocol questions considered here. Hazarika et al. (2022) replaced the 3 × 3 kernels of a U-Net with a bank of 1 × 1, 3 × 3, and 5 × 5 kernels and reported a DSC of 0.9700 on a subset of ADNI, using 508 training and 1500 test images; no subject-level separation between these sets is reported, so the same risk of same-subject slices crossing the split there as well, and the very high DSC obtained should be read with this in mind [62]. Bhosale and Dixit (2026) compared U-Net, Attention U-Net, and two SegNet variants on 2D ADNI hippocampal slices using a shared hybrid BCE/Dice loss [63]. They reported that Attention U-Net achieved a lower mean validation Dice (0.9589) than the standard U-Net (0.9655). This finding is consistent with the relative performance observed in our experiments, where the re-implemented Attention U-Net underperformed the U-Net across all three datasets. Although excessive suppression of foreground features by attention gating may contribute to this behavior, the available results do not conclusively establish this explanation. Two further studies illustrate the same efficiency contrast from the opposite direction: Yang et al. (2025) added a residual feature-reconstruction block, an efficient attention pyramid, and a dynamic frequency-context module to U-Net++ (FED-UNet++) and reported a DSC of 0.9012 on the Decathlon hippocampus task [64], and Liu et al. (2026) combined four attention and edge-refinement modules (ESCFR-Net) to reach a DSC of 0.9004 on a clinical MCI cohort [65].

## 2. Materials and Methods

### 2.1. Datasets

Three public datasets of 3D brain MRI were used in this study. All three datasets included de-identified secondary data released for research purposes. To maintain a direct comparison with published results, patch extraction and slicing followed the conventions used in the studies with which we compared [11,38]. Because the hippocampus occupies a very small fraction of each slice, all three datasets were strongly class-imbalanced; therefore, slice interpolation augmentation was applied to the training data, as described below. Table 1 summarizes the datasets.

**Figure 1:**
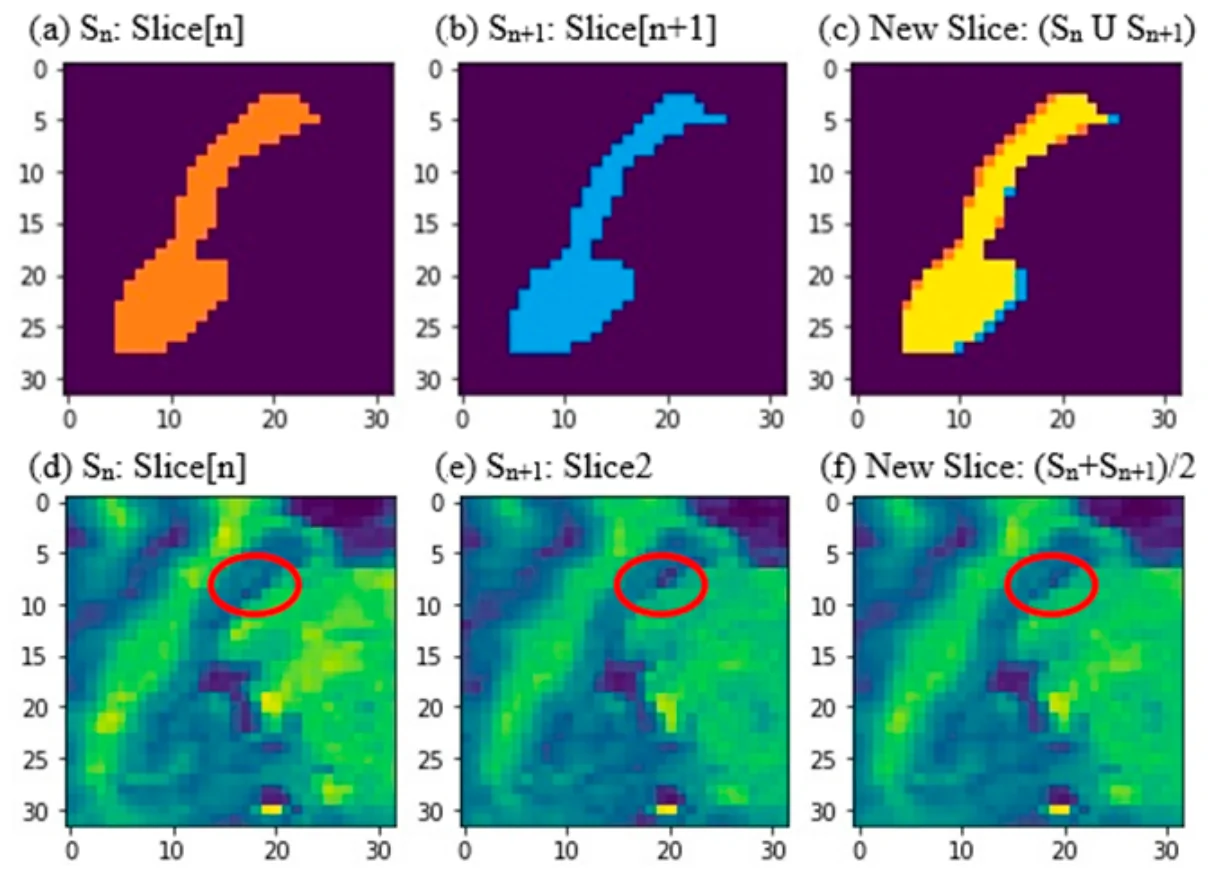
Slice-interpolating augmentation illustrated on the Decathlon dataset. (**a**,**b**) Two consecutive label slices, shown in orange and blue; (**c**) the generated label, the union of (**a**,**b**); (**d**,**e**) the corresponding image slices; (**f**) the generated image, the arithmetic mean of (**d**,**e**). Differences are highlighted in red.

Augmentation generated one additional slice from each pair of consecutive labelled slices. The new label was the union of the two source labels, and the new image was their voxel-wise arithmetic mean. Figure 1 illustrates the procedure: panels (a) and (b) show two consecutive label slices in orange and blue, and panel (c) their union; and panels (d) and (e) show the corresponding image slices, and panel (f) their mean, with the regions that differ marked in red. Test slices were never augmented, so that the reported scores refer to non-augmented data only. For every dataset and method, including the baselines, the held-out test slices were drawn from the same pre-cropped patches as the training and validation slices; no evaluation was performed on uncropped slices. This is one augmentation strategy among several that have been compared for medical image segmentation, and the choice is known to affect accuracy materially [66]. It was compared against two alternatives under the subject-grouped protocol of Section 2.6. on ADNI, using FDNetX2 with the original hybrid loss and 80 training epochs: a light geometric transform (random rotation up to ±7°, translation up to ±4% of the slice size, and isotropic scaling in 0.95–1.05, applied online to training slices with probability 0.5, without generating extra samples), and the same geometric transform combined with a random intensity perturbation (gamma, multiplicative gain, and additive noise). The mean subject-level validation Dice across the five development folds was 0.8928 with the slice-interpolation scheme described above, 0.8999 with geometric augmentation alone, and 0.8998 with geometric plus intensity augmentation—the geometric transform improved on slice-interpolation by about 0.007 DSC, while adding intensity perturbation gave no further gain. Patch oversampling of the hippocampal region was not evaluated and remains for future work.

#### 2.1.1. ADNI

Data used in the preparation of this article were obtained from the Alzheimer’s Disease Neuroimaging Initiative (ADNI) database (adni.loni.usc.edu). ADNI was launched in 2003 as a public–private partnership led by Principal Investigator Michael W. Weiner, MD. Its primary goal has been to test whether serial MRI, positron emission tomography, other biological markers, and clinical and neuropsychological assessment can be combined to measure the progression of mild cognitive impairment and early Alzheimer’s disease.

From the ADNI LONI Image Data Archive, we obtained 322 T1-weighted volumes of 166 × 256 × 256 voxels with accompanying hippocampal masks acquired from 181 women and men aged 55–91 years with Alzheimer’s disease, mild cognitive impairment, or normal cognition; several participants contributed more than one scan acquired at different time points. A random half of the volumes (161) was retained to limit computational cost, and a 96 × 96 × 96 patch covering both hippocampi was cropped around the volume center. Sagittal slicing yielded 96 slices of 96 × 96 pixels per volume, that is, 15,456 slices in total, of which 5295 contained labelled voxels. The labelled class occupied 0.38% of the total slice area before augmentation and 0.62% after augmentation.

#### 2.1.2. Medical Segmentation Decathlon

The hippocampus task of the Medical Segmentation Decathlon [67] comprises 394 T1-weighted volumes of different dimensions, manually labelled at the Vanderbilt University Medical Center, and divided by the organizers into 263 training and 131 test cases, whose labels were withheld. Only the labelled training cases could be scored outside the challenge, and 260 of them were used here. The task defined two foreground labels, the anterior and posterior hippocampus, which were merged into a single hippocampal class for this study. Volumes were resampled to a common 32 × 32 × 32 grid, and contiguous sagittal slices containing hippocampal voxels were retained, following the protocol of Huang et al. [38]. This yielded 2080 slices of 32 × 32 pixels, increasing to 4160 after augmentation. The labelled class occupied 12.75% of the total slice area before augmentation and 13.52% after augmentation, which was an order of magnitude more than in the other two datasets because the volumes were tightly cropped around the structure.

#### 2.1.3. IBSR

The Internet Brain Segmentation Repository (IBSR), distributed through the Neuroimaging Tools and Resources Collaboratory (https://www.nitrc.org/projects/ibsr), provides 18 volumes of 128 × 256 × 256 voxels with expert segmentations of 43 brain structures, of which only the hippocampal labels were used. A 96 × 96 × 120 patch covering both hippocampi was cropped around the volume center, and sagittal slicing yielded 2160 slices of 96 × 96 pixels, rising to 3241 after augmentation. The labelled class occupies 0.51% of the total slice area before augmentation and 0.71% after it.

The held-out test slices are drawn from the same cropped patches as the training and validation slices (96 × 96 × 96 for ADNI and IBSR, 32 × 32 × 32 for Decathlon, Section 2.1.1, Section 2.1.2 and Section 2.1.3); “original” in the preceding sentence refers to the exclusion of augmented (slice-interpolated) data, not to evaluation on uncropped full slices. No model in this study is evaluated on an uncropped slice, and the hippocampus-localization step that a full-slice pipeline would require is therefore not exercised by any of the methods compared here, including the baselines. This simplifies the task relative to a full-slice or full-volume deployment, a point noted in the Limitations (Section 5).

### 2.2. Feature-Dominated U-Net

In the encoder of a U-Net, pooling progressively discards spatial resolution, so that features without spatial extent—corners, thin protrusions, and internal folds—are attenuated as the depth increases; at the deepest level, only large, spatially coherent regions survive. Skip connections exist to restore this information. In the decoder, transposed convolution enlarges the coarse representation, and the encoder feature map is concatenated with it. Nevertheless, restoration is incomplete because a single concatenation gives the detailed encoder features a limited share of the input to the following convolutions: fine detail and upsampled context enter in a ratio of one to two. Two failure modes followed. Detail that is not recovered raises the false-negative (FN) count; conversely, the upsampled coarse representation tends to spread beyond the true boundary, raising the false-positive (FP) count. The two are coupled, so reducing one at the expense of the other does not necessarily improve the overlap.

We therefore increase the share of the encoder feature map by concatenating several identical copies of it. Writing D for the upsampled decoder feature map, *E* for the encoder feature map arriving on the skip connection, *E_i_* for its *i*-th copy and n for the number of copies, the concatenation performed at each decoder level is

(1) $$F_{\mathrm{dom}}\left(D,E,n\right)=D+\sum_{i=1}^{n}w_{i}.E_{i}$$

where w_i_ weighs the *i*-th copy. In all experiments reported in this paper the copies are unweighted, that is *w_i_ =* 1 for every *i*, so that Equation (1) reduces to

(2) $$F_{\mathrm{dom}}\left(D,E,n\right)=D+\sum_{i=1}^{n}E_{i}$$

The mechanism introduces no new learnable component—no gate, no transform, no additional sub-network—and the copies themselves carry no weights; the only parameter change is that the convolution immediately following each concatenation must have more input-channel kernel slices to accommodate the wider input, which is the modest, linear growth already quantified in Section 3.6 (48,384 parameters per additional copy) rather than an independent learnable mechanism. Each repeated copy is associated with a separate set of convolutional kernel weights, allowing the same encoder features to contribute through multiple parameter pathways. This alters the parameterization and optimization dynamics of the encoder branch without introducing a separate attention or gating module. Making *w_i_* learnable is a natural extension and is discussed in Section 4; however, it was not used in this study.

The convolution immediately after each concatenation uses the standard convolution weight layout, in which a separate kernel slice exists for every input channel. No new filters or filter copies are introduced, and the same output-channel count is used regardless of n. Concretely, for the first decoder level of FDNetX2 (*n* = 2 copies of the 64-channel encoder map E, concatenated with the 64-channel upsampled map *D* into a 192-channel tensor [*E*, *D*, *E*]), the following 3 × 3 convolution with 64 output channels already has one kernel slice *W*_*E*1_, *W_D_*, *W*_*E*2_ ∈ *ℝ*^{3×3×64×64}^ for each of the three 64-channel blocks, exactly as it would for a 192-channel input from any other source. This is standard convolution bookkeeping and requires no architectural change beyond accepting more input channels. The output is a single sum over all input channels, as in any convolution: because E’s two copies are identical, the two kernel slices that see them, W_E1_ and W_E2_, always receive the same input and can be replaced after training by their sum (*W*_*E*1_ + *W*_*E*2_) acting once on a single copy of *E*, without changing the layer’s output. This is the sense in which Section 2.2 states that repetition does not enlarge the family of representable functions: the extra input channels change how many parameters see the encoder detail during training and at initialization (Section 2.2, paragraph on Glorot initialization), not how the convolution combines its inputs or how many distinct functions it can compute.

Increasing n moves the detail-to-context ratio towards balance—one to two at *n* = 1, two to three at *n* = 2, and three to four at *n* = 3—but also multiplies redundant input channels, which is expected to promote overfitting beyond some point. Therefore, the optimal degree of domination is an empirical question, and we evaluated three settings: FDNetX2, FDNetX3, and FDNetX4, with *n* = 2, 3, and 4, respectively.

**Figure 2:**
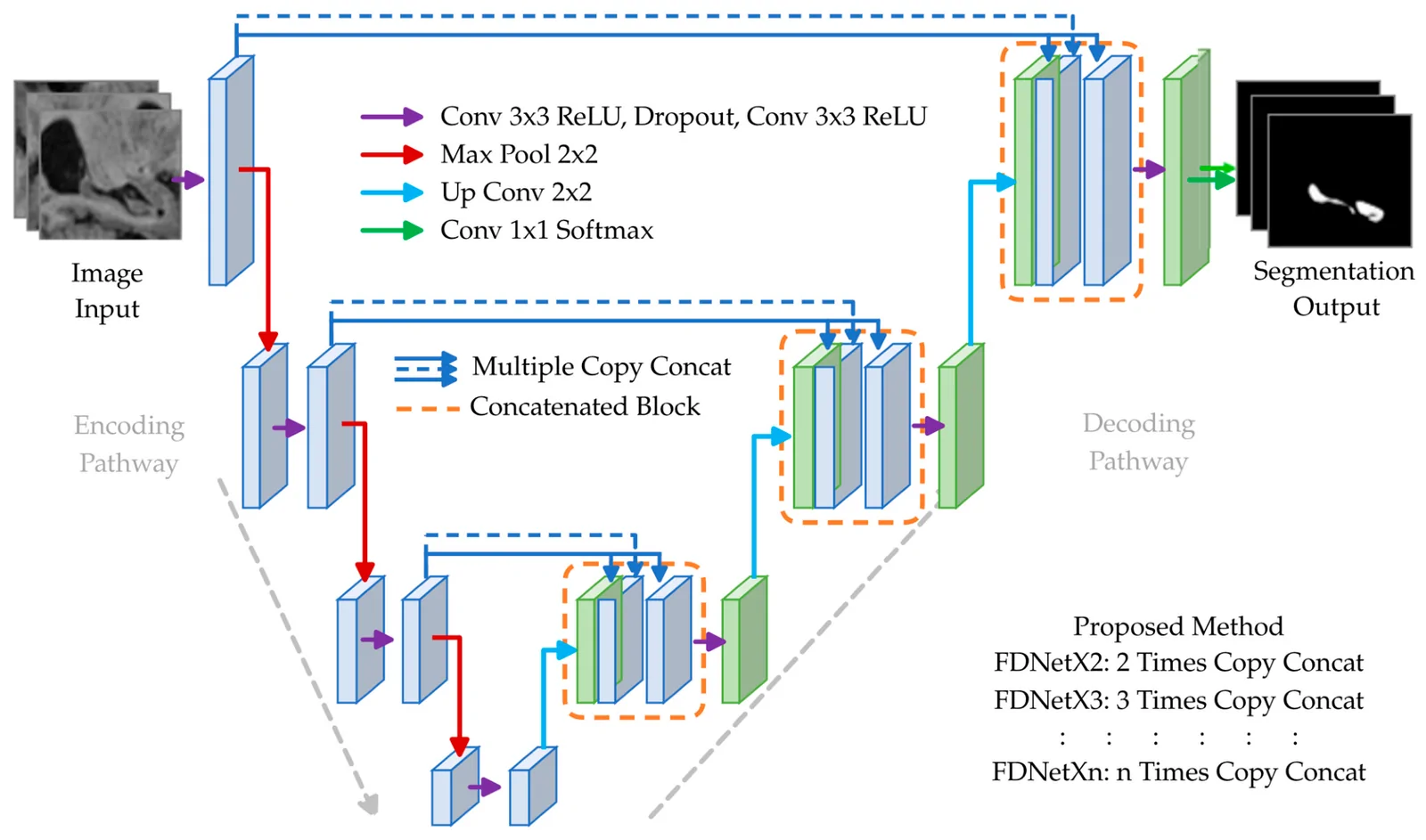
Architecture of the proposed network, drawn for FDNetX2, in which the encoder feature map is concatenated twice at each skip connection. The same template applies to FDNetX3 and FDNetX4, with three and four copies, respectively.

Figure 2 shows the resulting architecture for *n* = 2; the template is identical for other values of n, with the number of concatenated copies changed. Each encoder level applies convolution, dropout, convolution, and max pooling, with ReLU activation in the convolutions. Each decoder level mirrors this, replacing max pooling with a transposed convolution (Conv2DTranspose) and concatenating the encoder copies as described above. A final convolution with softmax activation produces the predicted mask at the original slice resolution.

**Figure 3:**
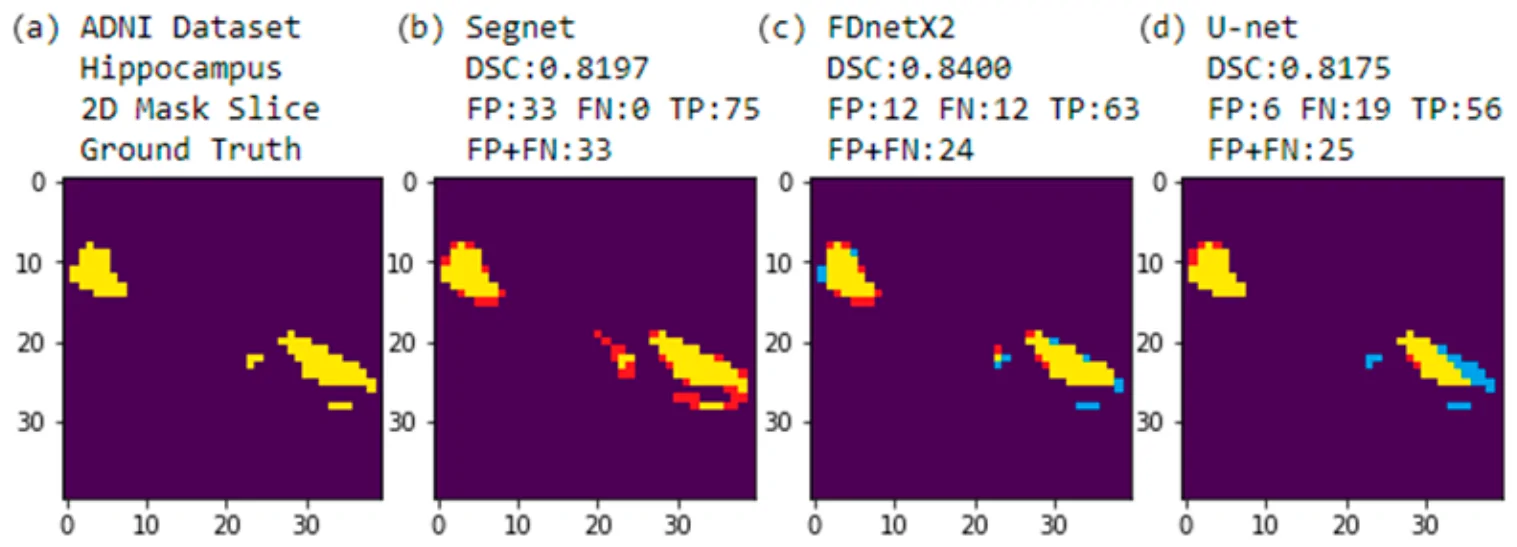
Relationship between FN, FP, and DSC on one ADNI test slice (true positives in yellow, FP in red, and FN in blue). (**a**) Reference mask; (**b**) SegNet, DSC 0.8197, FP = 33, FN = 0; (**c**) FDNetX2, DSC 0.8400, FP = 12, FN = 12; (**d**) U-Net, DSC 0.8175, FP = 6, FN = 19.

Figure 3 illustrates why the balance between the two error types, rather than either count alone, is the quantity of interest. On the slice shown, SegNet produced 33 FP and no FN pixels, and U-Net produced 6 FP and 19 FN pixels; their DSC values were almost equal (0.8197 and 0.8175). FDNetX2 produced 12 FP and 12 FN pixels—the same total error as U-Net to within one pixel—yet reached a higher DSC of 0.8400, because the smaller FN count leaves a larger true-positive count. Therefore, the objective is to reduce FN and FP together, which is what feature domination is designed to achieve: recovering detail suppresses FN, while retaining a strong detail signal in the decoder limits the spreading that produces FP.

Because the encoder feature maps are repeated identically, their contributions to the convolution following concatenation can be expressed through the sum of the corresponding kernels. Specifically, n identical copies of an encoder feature map E, associated with convolutional kernels *W*_1_, …, *W_n_*, are functionally equivalent to a single copy processed by the aggregated kernel *W*_1_ + … + *W_n_*. Therefore, feature repetition does not expand the set of functions representable by the network; instead, it changes the parameterization and optimization of the encoder contribution. Under independent Glorot initialization, the variance of the aggregated encoder kernel increases with the number of copies. Relative to the decoder branch, this can increase the initial contribution of encoder features, with an expected scaling proportional to $\sqrt{n}$ under comparable input statistics. During training, identical feature copies produce identical gradients with respect to their corresponding kernel slices. Under standard gradient descent, their aggregated update scales with n, whereas the effective update under Adam additionally depends on adaptive moment normalization and cannot generally be assumed to scale linearly. Thus, feature domination is interpreted as an implicit modification of feature weighting and optimization dynamics rather than an increase in representational capacity. Increasing the number of copies may strengthen the contribution of fine-grained encoder information, but excessive repetition may introduce an unfavorable balance between encoder detail and decoder context. Consistent with this interpretation, the slice-level experiments in Section 3.1 identified *n* = 3 as the best-performing setting on ADNI and Decathlon and *n* = 2 on IBSR.

### 2.3. Baseline Methods

Three widely used segmentation networks, U-Net [21], Attention U-Net [23], and SegNet [24], were re-implemented and trained under the same protocol as FDNet. In addition, the discriminative feature network of Shi et al. [11] was included, and the published results for further methods evaluated on the same datasets are reproduced in Section 3.3 for context. Because Shi’s method applies its own augmentation scheme, in which one three-channel slice is formed from three single-channel slices, its test set differs in size from the others; this is visible in the per-slice distributions in Section 3.5.

### 2.4. nnU-Net 2D Baseline

An nnU-Net v2 (2.8.1) 2D baseline was additionally trained on ADNI. The dataset was converted to a dedicated nnU-Net [36] raw/preprocessed dataset (one T1-weighted MRI channel, binary hippocampus label, 96 × 96 patches) using the identical subject-grouped split of Section 2.6—the same 288 development volumes and the same fixed 34 held-out test volumes, with custom fold definitions reproducing the GroupKFold partition. nnU-Net’s own default 2D configuration, architecture, loss, optimizer, and data-augmentation policy were kept unchanged; a custom trainer limited each fold to 120 epochs to match the epoch budget of the subject-grouped FDNet runs. Predictions were aggregated per volume and then per subject using the same confusion-count definitions of Dice, foreground IoU, and two-class mean IoU used throughout this study (Section 2.7), so that the nnU-Net numbers were directly comparable with the FDNet and baseline numbers; held-out test labels were not used for model selection.

### 2.5. Training Configuration

All models were implemented in TensorFlow 2 and trained on a workstation with an Intel Core i9-11900K processor, 32 GB of RAM, and an NVIDIA GeForce RTX 3060 graphics card. Optimization was performed using Adam with an initial learning rate of 1 × 10^−4^ and a batch size of 16, which was chosen to fit the available GPU memory. Dropout with a rate of 0.2 was applied between the convolutions of every encoder and decoder block, as shown in Figure 2.

Training minimized the hybrid loss combining the Dice loss and binary cross-entropy. Cross-entropy alone is dominated by the background class when the foreground occupies a fraction of a percent of the slice, as it does in ADNI and IBSR (Table 1); the Dice loss optimizes the overlap directly and is less sensitive to this imbalance. The combination

*L* = α *L*_Dice_ + (1 − α) *L*_BCE_ (3)

Therefore, it optimizes the pixel-wise classification accuracy and regional overlap. The weighting factor was set to α = 0.5.

Each experiment used a five-fold cross-validation. Before augmentation, 10% of the original slices were randomly reserved as a held-out test set and were not used during the model training or augmentation. The remaining 90% of the slices were divided into five folds. The reported scores are the mean performances of the five resulting models evaluated on the same non-augmented test set.

### 2.6. Subject-Level Cross-Validation

The original slice-level random test split described in Section 2.5 does not guarantee that slices originating from the same MRI volume or from different scans of the same participant are assigned exclusively to a single data subset. This introduces a potential risk of subject-level data leakage and may lead to optimistic performance estimates. To address this limitation, an additional subject-grouped evaluation was performed on ADNI using all 322 available MRI volumes from 181 participants, rather than the randomly selected subset of 161 volumes used in the original slice-level experiments. Before slicing or augmentation, participants were divided into a fixed held-out test set comprising 19 subjects (34 volumes) and a development set comprising 162 subjects (288 volumes). Five-fold cross-validation was performed on the development set using GroupKFold, ensuring that all volumes belonging to the same participant remained within a single partition in each fold. The original FDNetX2 training configuration was first evaluated under this subject-grouped protocol to establish a reference result. Subsequent training modifications, including geometric augmentation, loss-function refinement, increased training duration, and the use of a 2.5D input comprising three consecutive sagittal slices, were assessed using development-fold validation performance. The final configuration was then applied to all three FDNet variants. The fixed held-out test subjects were evaluated only after model configuration, and hyperparameter selection had been completed and were not used during optimization or model selection. Consequently, the original slice-level results (Table 2) and the subject-grouped results (Table 3 and Table 4) represent distinct evaluation protocols and should not be interpreted as directly controlled comparisons of data-partitioning strategies alone.

### 2.7. Evaluation Metrics

Let Y denote the reference mask and Ŷ the predicted mask, and let TP, TN, FP, and FN denote the numbers of true positives, true negatives, false positives, and false negatives, respectively. The primary metric is the Dice similarity coefficient,

*DSC* = 2|*Y* ∩ *Ŷ*|/(|*Y*| + |*Ŷ*|) (4)

reported alongside the mean intersection over union (mIoU), which averages *IoU* = |*Y* ∩ *Ŷ*|/|*Y* ∪ *Ŷ*| over the semantic categories of the image [38], recall (sensitivity) = *TP*/(*TP* + *FN*), positive predictive value (PPV, precision) = *TP*/(*TP* + *FP*), and accuracy = (*TP* + *TN*)/(*TP* + *TN* + *FP* + *FN*). Recall and PPV are reported together because each ignores one error type: PPV is insensitive to FN and recall to FP; therefore, either can be made large at the expense of the other.

To quantify the balance between false-negative and false-positive predictions, we reported the mean number of misclassified pixels per slice for each type of error. Let m denote the number of evaluated slices and let *y_i_* and *ŷ_i_* represent the reference and predicted masks for slice *i*, respectively. The false-negative pixels per slice (FNPr) and false-positive pixels per slice (FPPr) are defined as follows:

(5) $$\mathrm{FNPr}=\frac{\sum_{i=1}^{m}\left[\left(y_{i}-{\hat{y}}_{i}\right)+\left|y_{i}-{\hat{y}}_{i}\right|\right]}{2m}$$

(6) $$\mathrm{FPPr}=\frac{\sum_{i=1}^{m}\left[\;\left({\hat{y}}_{i}-y_{i}\right)+\left|{\hat{y}}_{i}-y_{i}\right|\;\right]}{2m}$$

Accordingly, FNPr and FPPr represent the average numbers of false-negative and false-positive pixels per slice, respectively, rather than the conventional normalized false-negative and false-positive rates. Their sum, FNPr + FPPr, represents the mean total number of misclassified pixels per slice. These metrics complement DSC by characterizing the composition of segmentation errors and are used throughout Section 3 to examine the relationship between the error distribution and segmentation overlap.

The two rates are reported together because DSC depends on them asymmetrically. Writing |*Y*| = *TP* + *FN* for the area of the reference mask, which is fixed by the ground truth, Equation (4) can be rewritten as *DSC* = 2(|*Y*| − *FN*)/(2|*Y*| − *FN* + *FP*). At a fixed total number of misclassified pixels, this decreases as FN grows; therefore, of two predictions with the same error count on the same slice, the one that misses less of the structure necessarily scores higher. Differentiating the same expression shows that the marginal cost of a false negative exceeds that of a false positive by the factor (*TP* + *FN* + *FP*)/*TP*, which is never below one and grows as the cross-section of the structure shrinks. Therefore, reducing FP alone is the cheaper but less rewarding option, and it is the balance of the two rates rather than in isolation that determines the overlap.

Figure 3 provides an example. The three DSC values reported there are reproduced exactly by a reference area of 75 pixels: SegNet, with 33 FP and no FN, scored 150/183 = 0.8197; FDNetX2, with 12 of each, scored 126/150 = 0.8400; and U-Net, with 6 FP and 19 FN, scored 112/137 = 0.8175. FDNetX2 and U-Net differed by a single pixel in the total error, yet by 0.0225 in DSC, a difference attributable entirely to FN. On that slice, a false negative costs 38% more overlap than a false positive, which is why a method that suppresses the background, as the attention-gated baseline does, buys inexpensive reductions in FP at the price of expensive increases in FN.

Two qualifications apply. The relations above hold slice by slice, whereas the values reported in Section 3 are means: DSC is averaged over slices, while FNPr and FPPr average pixel counts; therefore, a lower FNPr + FPPr does not entail a higher mean DSC as a matter of algebra. This is why the per-slice distributions of Section 3.5 are reported alongside the aggregates rather than in place of them. In addition, slices whose reference mask is empty have an undefined DSC and are excluded from the averages.

### 2.8. Statistical Analysis

To assess whether the observed differences among the three FDNet variants and between FDNet and the baseline methods were statistically significant, paired subject-level comparisons were performed on the ADNI dataset using the subject-grouped evaluation protocol described in Section 2.6. All models were evaluated on the same fixed held-out test set comprising 19 subjects, enabling subject-wise pairing of the dice scores. Statistical significance was assessed using the two-sided Wilcoxon signed-rank test for paired samples. To account for multiple tests, the Holm–Bonferroni procedure was applied across the nine pairwise comparisons reported in Table 4, with an adjusted significance threshold of 0.05. In addition, 95% bootstrap confidence intervals for the mean subject-level Dice of each model were estimated using 10,000 subject-level resamples. The results indicated that all three FDNet variants significantly outperformed U-Net, Attention U-Net, and SegNet after multiple comparison correction (Holm-adjusted *p* < 0.0001). No statistically significant differences were observed among the FDNet variants (uncorrected *p* = 0.14–0.57). Although FDNet achieved slightly higher Dice scores than the nnU-Net ensemble, these differences were not statistically significant after the Holm correction. Statistical comparisons were restricted to the ADNI subject-grouped evaluation and should not be interpreted as evidence of statistical significance in the Decathlon or IBSR dataset.

## 3. Results

### 3.1. Segmentation Accuracy and Error Balance

Table 2 presents the DSC, FPPr, and FNPr for the three FDNet variants and the three re-implemented baselines on all three datasets. On every dataset, the highest DSC was obtained by an FDNet variant, and in each case, that variant also achieved the lowest combined mean per-slice error count (FNPr + FPPr) among the six methods. The three variants differed only in the number of copies, and the best setting was not the same across datasets: FDNetX3 gave the highest DSC on ADNI (0.9339) and Decathlon (0.9470), whereas FDNetX2 was best on IBSR (0.9477). In all three cases, FDNetX4 was worse than the best variant, consistent with the expectation that redundant copies eventually promote overfitting rather than improving the recovery of detail.

The error decomposition shows why overlap alone is an insufficient summary. Attention U-Net attains the lowest FPPr on ADNI (2.7284) and by a wide margin the lowest on IBSR (0.4992), yet records the lowest DSC on both, because its FNPr is the highest of any method on both datasets (4.6489 on ADNI and 12.0235 on IBSR, against 1.43–1.80 and 0.38–1.66 for the remaining methods): it under-segments, and the pixels it does label are almost all correct. SegNet shows the mirror image on IBSR, with the lowest FNPr (0.3807) but an FPPr of 4.8311, and its DSC of 0.9464 remains below that of FDNetX2 (0.9477) and FDNetX3 (0.9471) despite the more favorable FN count. No FDNet variant holds the lowest FPPr on any dataset—that value belongs to Attention U-Net on all three—and where an FDNet variant does hold the lowest FNPr (FDNetX4 on ADNI, FDNetX2 on Decathlon) it is not the variant that achieves the highest DSC. The best-scoring variant holds the lowest sum FNPr + FPPr, and it is this quantity that the DSC ranking follows.

### 3.2. Subject-Grouped Evaluation and Statistical Comparison

The results presented in Table 2 were obtained using a random slice-level test split in which slices from the same subject could appear in both the training and test sets. To address the potential subject-level leakage associated with this partitioning strategy, a subject-grouped re-evaluation was conducted, as described in Section 2.6 of this paper., ensuring that subjects were separated across the training, validation, and test partitions. The evaluation also included a nnU-Net v2 2D baseline trained under the same subject-grouped partitioning scheme (Section 2.4.), which achieved a mean subject-level validation Dice of 0.8999 across five development folds on ADNI. The corresponding subject-level results and statistical comparisons are presented in Table 3 and Table 4, following the statistical-testing protocol described in Section 2.8.

Under the subject-grouped protocol with the original training configuration, FDNetX2 achieved a mean subject-level validation Dice of 0.8863 across the five development folds and a Dice of 0.8771 on the fixed held-out test set, compared with 0.9321 under the original slice-level split (Table 2). This difference illustrates the potential optimism associated with slice-level evaluation, although it cannot be attributed exclusively to subject overlaps without controlling for other protocol differences in the study. Targeted modifications assessed exclusively on the development folds—including geometric augmentation, a hybrid cross-entropy/soft-Dice loss, extending training from 80 to 120 epochs, and incorporating a 2.5-dimensional input comprising three consecutive sagittal slices—progressively increased the mean subject-level validation Dice of FDNetX2 from 0.8863 to 0.9069. Applying this final configuration to FDNetX3 and FDNetX4 yielded validation Dice scores of 0.9070 and 0.9069, respectively, indicating a closely matched performance among the three variants.

On the fixed held-out test set, FDNetX2, FDNetX3, and FDNetX4 achieved subject-level Dice scores of 0.9047, 0.9050, and 0.9044, respectively, exceeding those of the nnU-Net five-fold ensemble (0.9019), U-Net (0.8866), Attention U-Net (0.8849), and SegNet (0.8243), as shown in Table 3. The maximum difference among the FDNet variants was only 0.0006 Dice, indicating that increasing the repetition factor beyond two offered no additional benefit under the optimized subject-grouped configuration. Although nnU-Net achieved the highest test precision (0.9147), its recall (0.8939) was lower than that of the leading FDNet variants, which is consistent with the false-positive/false-negative trade-off discussed in Section 4. SegNet exhibited substantial across-fold variability (SD = 0.053), driven primarily by one development fold with a Dice score of 0.73, despite apparently normal training convergence. Excluding this fold would yield a four-fold mean of 0.847, which is still below that of the FDNet variants and U-Net; however, all primary results retained the complete five-fold evaluation to avoid selective exclusion.

Pairwise Wilcoxon signed-rank tests on the held-out test subjects demonstrated that each FDNet variant significantly outperformed U-Net, Attention U-Net, and SegNet (Holm-adjusted *p* < 0.0001; Table 4). No statistically significant differences were detected among the three FDNet variants (*p* = 0.14–0.57), whereas their numerical advantage over the nnU-Net ensemble was significant before, but not after, Holm correction. The complete statistical results, including 95% bootstrap confidence intervals, are reported in Table 4. Overall, these findings demonstrate that FDNet retains its performance advantage over the re-implemented baselines under subject-grouped evaluation, while its superiority over nnU-Net remains statistically unconfirmed after multiple-comparison correction. The slice-level results in Table 2 are retained to provide context for previously published studies employing similar evaluation strategies, whereas the subject-grouped results offer a more rigorous assessment of generalization to unseen subjects.

When the statistical results are considered, two conclusions can be drawn. First, every FDNet variant significantly outperformed all three re-implemented baselines on the held-out test set (*p* < 0.0001, surviving Holm correction). Second, the three FDNet variants were statistically indistinguishable from one another (*p* = 0.14–0.57); therefore, the choice of n is not supported as a significant factor on this test set, and the variants are best read as a family. FDNet also exceeded the nnU-Net ensemble by a small margin that was significant without correction (*p* < 0.03) but did not survive Holm correction across nine comparisons; therefore, FDNet is described as comparable-to-slightly-better than nnU-Net rather than decisively superior—achieved with one to two orders of magnitude fewer parameters.

To further assess the computational efficiency, Table 5 compares FDNet with the baseline architecture in terms of convolutional FLOPs and peak GPU memory usage under width-matched conditions, whereas the corresponding parameter counts are reported separately in Section 3.6. The conv-GFLOPs of the FDNet variants were comparable to those of U-Net and Attention U-Net (0.78–1.03 versus 0.85–0.89), rather than consistently lower. Their peak training memory usage is also higher (1.76–2.06 GiB versus approximately 1.39–1.41 GiB), as repeating the encoder feature map increases the width of the post-concatenation activation map. Nevertheless, all three architectures required substantially less memory than SegNet (5.01 GiB). Therefore, the efficiency advantage of FDNet at this width primarily lies in its reduced parameter count and, relative to SegNet, its lower computational cost. Combined with the statistically significant improvements in segmentation accuracy reported in Table 3 and Table 4, these findings indicate that FDNet achieves a favorable balance between model compactness and segmentation performance, rather than uniformly reducing computational operations or activation memory.

### 3.3. Comparison with Existing Hippocampus Segmentation Methods

Table 6, Table 7 and Table 8 place the results in the context of previously published work on the same three datasets. The scores for the methods re-implemented here were computed on the original, non-augmented test slices; scores attributed to other authors are those reported in the corresponding papers, and the protocols behind them differ in slice orientation, volume count, and preprocessing; therefore, the comparison is indicative rather than strictly controlled. The only exception among our own runs is Shi’s method, which uses its own three-channel augmentation and, therefore, a differently sized test set.

A further group of recent studies reported hippocampal DSC on ADNI or Decathlon but could not be placed in Table 6, Table 7 and Table 8 without misleading the reader because the quantity being averaged was not the same: some scored whole volumes, one scored substructures separately, one stratified by diagnostic group, and, for two, the evaluation unit was not stated in the accessible record. These are listed in Table 9, with the reason for each case, so that the omission is explicit rather than silent.

On Decathlon and IBSR, the highest DSC in the table was obtained by an FDNet variant, exceeding the closest published result by 0.0159 on Decathlon (0.9470 versus 0.9311 [38]) and 0.0284 on IBSR (0.9477 versus 0.9193 [38]). On ADNI, the situation changed while this work was being prepared: FDNetX3 improved on the best result published up to 2023 by 0.0103 (0.9339 versus 0.9236 [38]), but the attention-based CSDA-UNet reported 0.9512 on the same dataset [59], which was 0.0173 higher than that of FDNetX3. This model couples spatial attention with an inter-slice attention module and is trained on a considerably larger slice set (18,900 training slices from 100 subjects, against 15,456 slices from 161 volumes here); therefore, the two numbers were not produced under matched conditions. Nevertheless, the comparison shows that FDNet is not the most accurate method reported in ADNI. The ranking by PPV differed from that by DSC, as expected. On ADNI, Attention U-Net has a higher PPV (0.9217) than any FDNet variant, and on IBSR, its PPV of 0.9859 is the highest in the table, in both cases because PPV disregards the large FN counts reported in Table 2. Recall behaved symmetrically, with SegNet reaching 0.9918 on the IBSR against an FPPr of 4.8311. Accuracy was near-saturated on ADNI and IBSR (≥0.9987 for every model trained here, except Shi’s method, which was evaluated on a differently augmented test set) because the background class occupies more than 99% of each slice; therefore, it carries little information for this task and is reported only for comparison with earlier studies.

To allow a comparison under the slice-level conditions of Table 6, nnU-Net was also trained on the original slice-level split (an isolated dataset reproducing the exact 1529-slice ADNI test set used for the FDNet slice-level numbers) and evaluated with identical pooled-confusion metrics: it reached a DSC of 0.9332 (recall 0.9549, PPV 0.9124, FNPr + FPPr 4.79 pixels/slice), placing it between FDNetX2 (0.9321) and FDNetX3 (0.9339)—closely comparable in overlap, but obtained with a self-configuring network one to two orders of magnitude larger than the 0.53–0.63 M-parameter FDNet variants. On the subject-grouped held-out set, the three FDNet variants slightly exceeded the nnU-Net ensemble (0.9044–0.9050 versus 0.9019); the paired difference was significant before but not after Holm correction (Table 4), so FDNet is described as comparable to, or slightly better than, nnU-Net rather than decisively superior. The nnU-Net row is included in Table 6.

### 3.4. Qualitative Results

**Figure 4:**
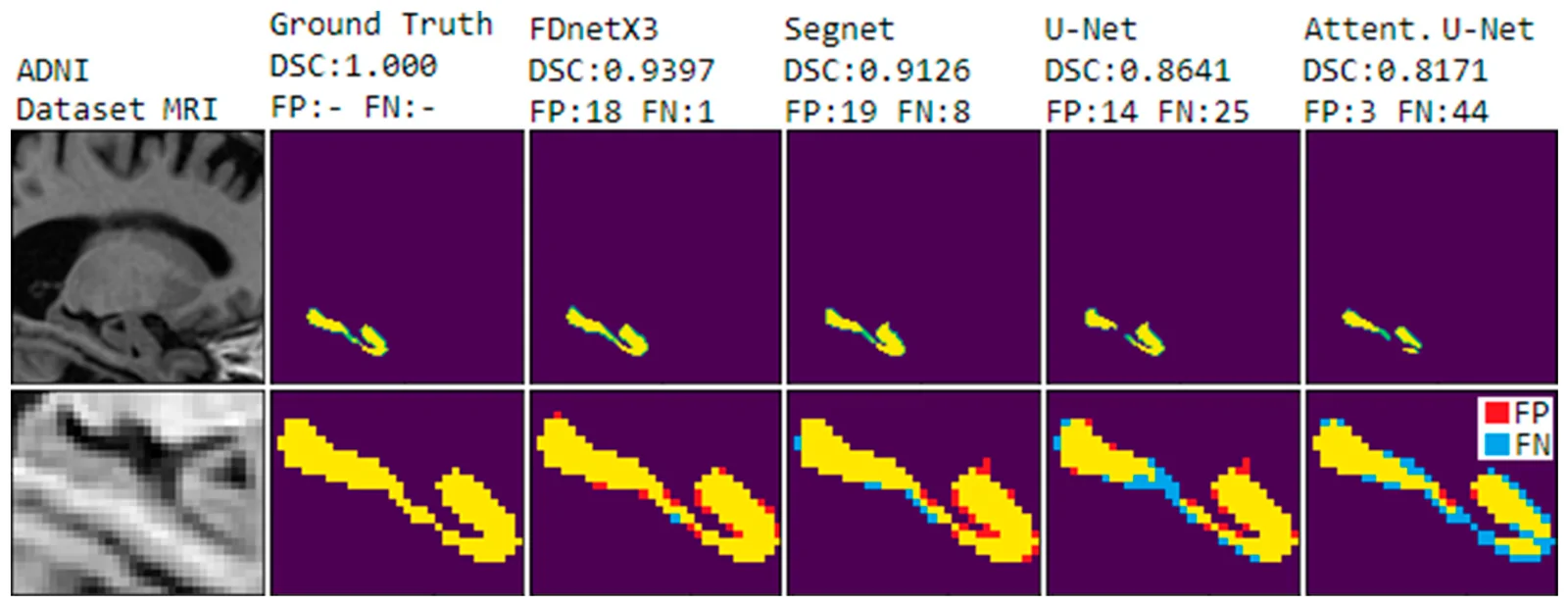
Test slice from the ADNI dataset segmented by the reference mask, FDNetX3, SegNet, U-Net, and Attention U-Net. FP pixels are red, FN pixels are blue and ground truth pixels are yellow. The hippocampal region is enlarged beneath each panel.

**Figure 5:**
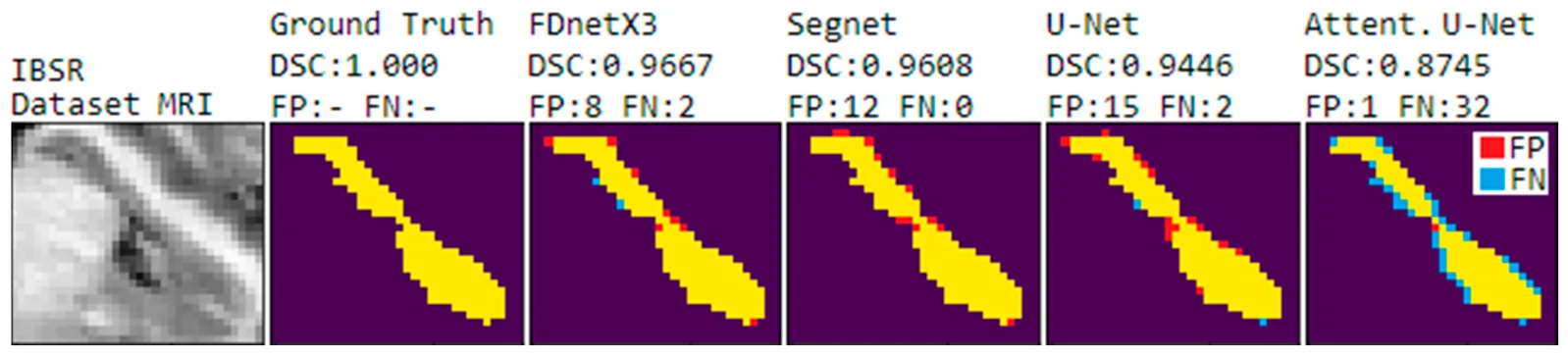
A test slice from the IBSR dataset segmented by the reference mask, FDNetX3, SegNet, U-Net, and Attention U-Net. FP pixels are red, FN pixels are blue and ground truth pixels are yellow.

**Figure 6:**
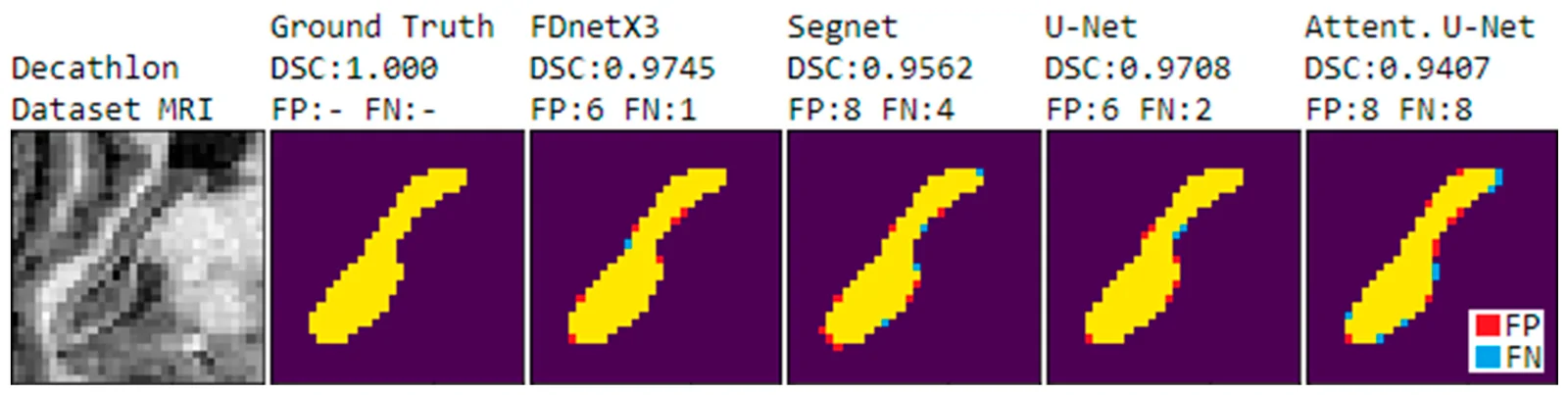
Test slice from the Medical Segmentation Decathlon dataset segmented by the reference mask, FDNetX3, SegNet, U-Net, and Attention U-Net. FP pixels are red, FN pixels blue and ground truth pixels are yellow.

Figure 4, Figure 5 and Figure 6 show one test slice from each dataset, segmented by FDNetX3 and the three baselines, with FP pixels in red, FN pixels in blue, and the DSC, FP, and FN values for that slice given above each panel. In Figure 4, the hippocampal region is additionally shown enlarged. Across the three examples, the FDNet output carried fewer and more evenly distributed erroneous pixels than the baselines: SegNet and U-Net showed red margins where the segmentation extended into the neighboring tissue, while Attention U-Net showed extended blue regions where the structure was missed, with 44 FN pixels on the ADNI slice and 32 on the IBSR slice. These single slices are illustrative and were selected to show the error patterns discussed above; they are not a substitute for the distributions reported in Section 3.5.

### 3.5. Per-Slice Distributions

**Figure 7:**
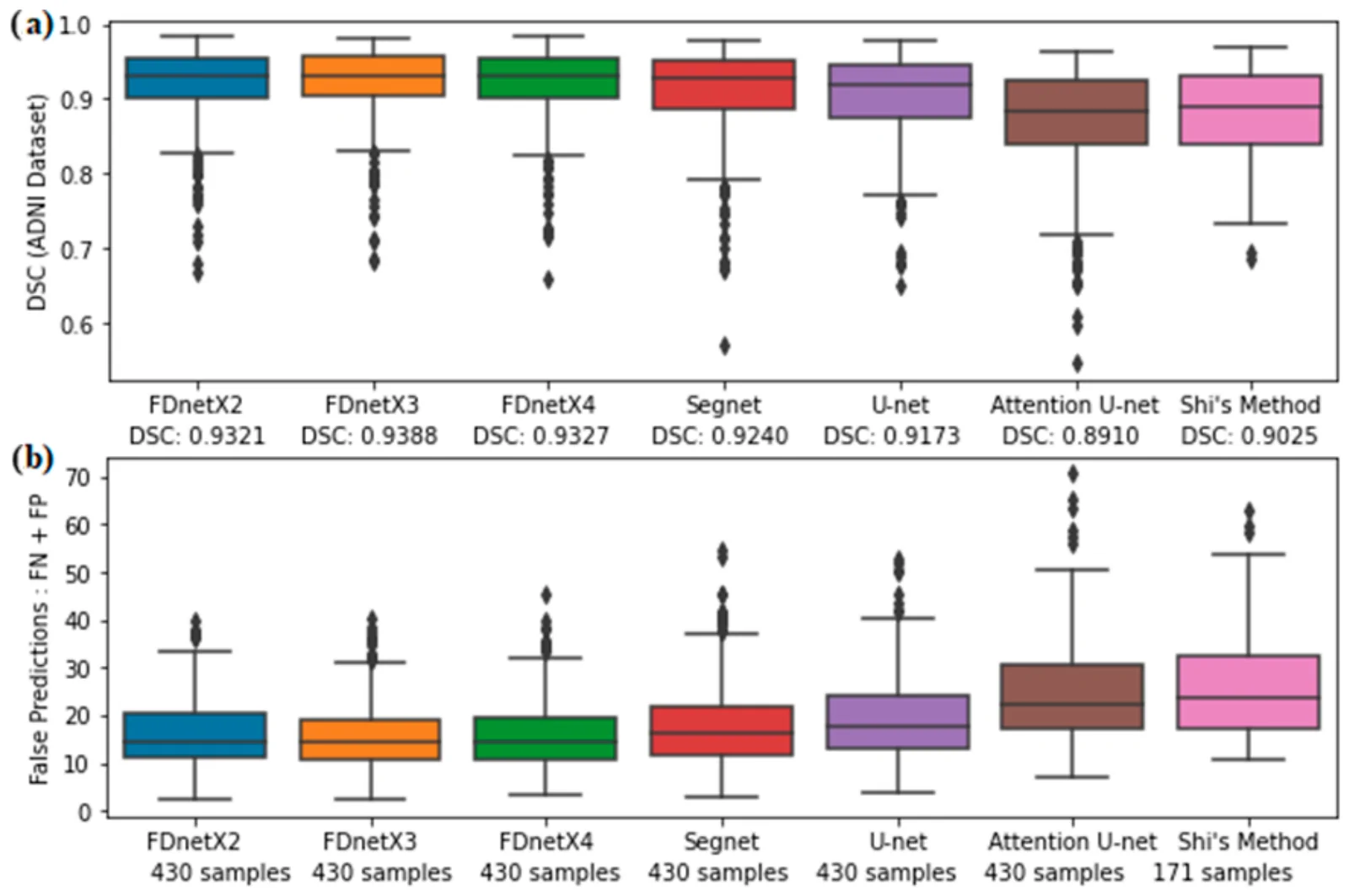
Per-slice distributions on the ADNI test set: (**a**) DSC; (**b**) total number of erroneous pixels (FN + FP).

**Figure 8:**
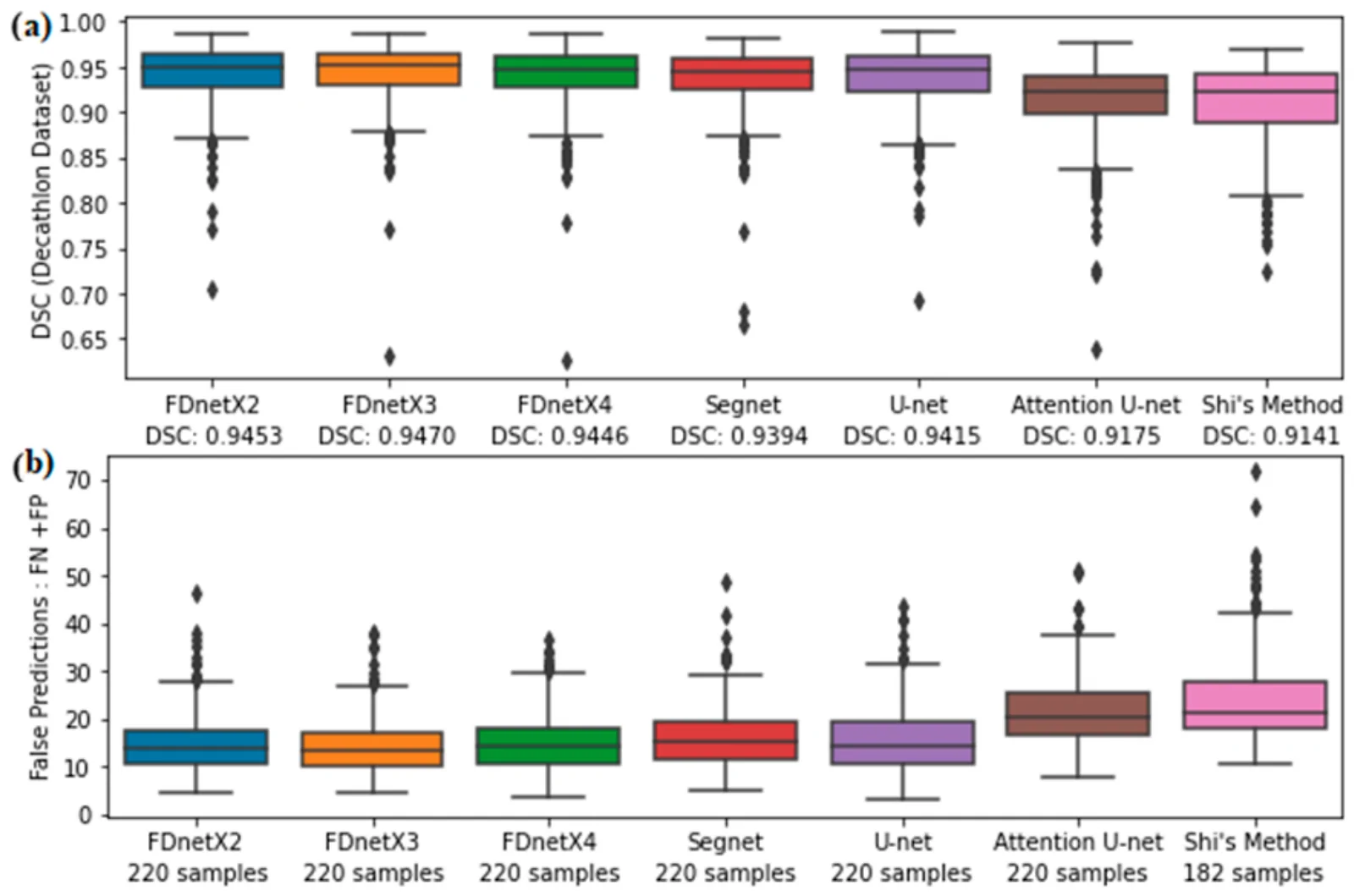
Per-slice distributions on the Medical Segmentation Decathlon test set: (**a**) DSC; (**b**) total number of erroneous pixels (FN + FP).

**Figure 9:**
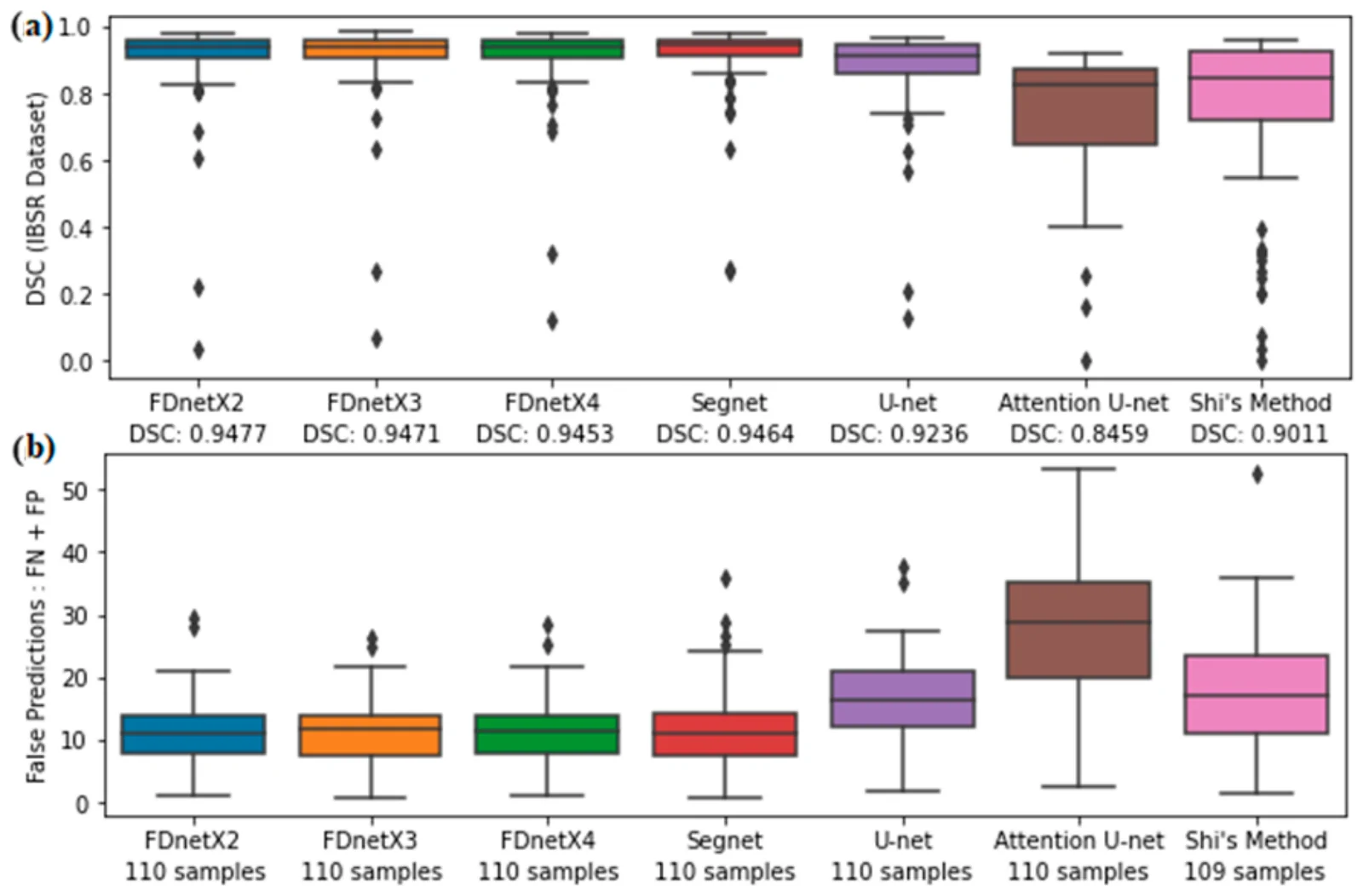
Per-slice distributions on the IBSR test set: (**a**) DSC; (**b**) total number of erroneous pixels (FN + FP).

Figure 7, Figure 8 and Figure 9 show, for each dataset, the distribution of the DSC and the total number of erroneous pixels over the test slices. The two panels of each figure are close to mirror images, confirming at the level of individual slices the relationship established in aggregate in Table 2: slices on which a method accumulates more erroneous pixels are the slices on which its overlap is lower, and the ordering of the methods by median DSC follows the ordering by median error count. The ordering of the FDNet variants matches Table 2 in each case: FDNetX3, FDNetX4, FDNetX2 on ADNI; FDNetX3, FDNetX2, FDNetX4 on Decathlon; and FDNetX2, FDNetX3 on IBSR, where SegNet is third. The distributions of the three variants overlap substantially on all three datasets, which should be considered when interpreting the differences between them (Section 4). The number of test slices for Shi’s method differs from that of the other methods for the reason given in Section 2.3.

### 3.6. Model Size and Computational Efficiency

Table 10 lists the parameter count and size of the stored weight file for each method. The FDNet variants use between 530,146 and 626,914 parameters, which is one to two orders of magnitude fewer than the baselines: 65 times fewer than U-Net, 70 times fewer than Attention U-Net, and 166 times fewer than SegNet. Within the FDNet family, the only cost of increasing the degree of domination is the additional input channels at each concatenation. This cost is strictly linear in the number of copies, and the measured counts bear this out: the increment between consecutive variants is exactly constant at 578,530 − 530,146 = 626,914 − 578,530 = 48,384 parameters, giving P(n) = 433,378 + 48,384n and implying a single-copy network of 481,762 parameters. The increment is also consistent with the layer geometry, since 48,384 = 9(16^2^ + 32^2^ + 64^2^): with 3 × 3 kernels, three skip connections carrying 16, 32, and 64 channels into equally wide convolutions reproduce it exactly, and among sets of power-of-two widths this factorization is unique. Raising the degree of domination from two copies to four therefore increases the parameter count by 18%, against the 65- to 166-fold gap that separates every variant from the baselines.

In addition, the floating-point operation counts were reported, computed directly from the TensorFlow graph of each model for a single 96 × 96 × 1 input using the TensorFlow profiler to provide an implementation-independent measure of computational cost alongside hardware-dependent timings. The parameter counts recomputed exactly matched Table 10 (530,146; 578,530; 626,914), confirming the architecture definitions. The corresponding operation counts were 0.776 GFLOPs (FDNetX2), 0.904 GFLOPs (FDNetX3), and 1.031 GFLOPs (FDNetX4) per slice, i.e., 0.388, 0.452, and 0.516 GMACs, respectively. A width-matched efficiency comparison, including conv-GFLOPs and peak training memory for the re-implemented baselines, is given in Table 5 for the subject-grouped setting. The values in Table 10 characterize the baselines at their original published widths.

The reduction in the parameter count was also reflected in the computational time. On the ADNI experiments, FDNetX2, FDNetX3, and FDNetX4 required approximately 8.90, 9.73, and 10.83 s per training epoch, respectively, compared with 50.98 s for U-Net, 66.59 s for Attention U-Net, and 79.28 s for SegNet. Inference showed the same trend: the FDNet variants required approximately 0.70–0.99 ms per slice, whereas U-Net, Attention U-Net, and SegNet required 2.11, 2.50, and 3.03 ms per slice, respectively. These measurements indicate that the reduction in model size is accompanied by a substantial reduction in actual computational time rather than representing parameter efficiency alone.

**Figure 10:**
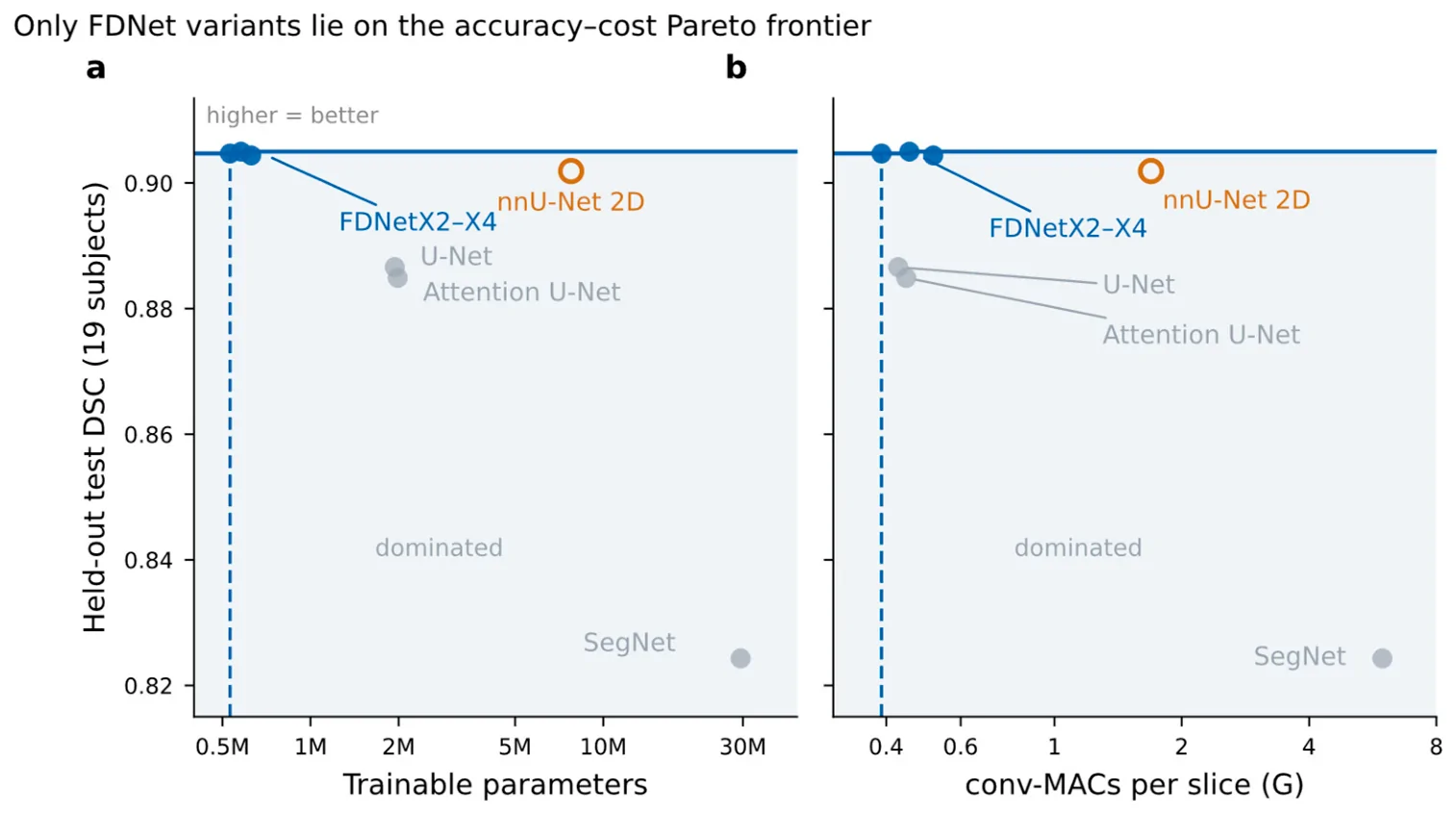
Accuracy–cost trade-off under the subject-grouped ADNI protocol. Held-out test DSC (19 subjects) is plotted against (**a**) the number of trainable parameters and (**b**) convolutional MACs per slice, both on logarithmic axes. The staircase marks the Pareto frontier (highest DSC at the lowest cost), and the shaded area is the dominated region. The dashed vertical line marks the parameter count (**a**) and MACs per slice (**b**) at which the FDNet variants reach the highest DSC, indicating the lowest-cost point on the Pareto frontier.

Figure 10 summarizes this trade-off in a single view, plotting the held-out test DSC of the subject-grouped evaluation against the parameter count and against the convolutional MACs per slice. In both panels, the Pareto frontier consists exclusively of FDNet variants, and no competing model attains the DSC of the FDNet family at any parameter or operation budget. The width-matched U-Net and Attention U-Net require 3.7 times more parameters at essentially the same operation count (1.1 times) yet score 0.018 and 0.020 DSC lower; nnU-Net, with 14.6 times the parameters and 4.3 times the operations of FDNetX2, remains 0.003 DSC below the best FDNet variant; and SegNet requires 55.6 times the parameters and 15.3 times the operations for a DSC 0.081 lower. Within the FDNet family itself, FDNetX4 is dominated by FDNetX3, although the difference between them is not statistically significant (Table 4). The accuracy obtained from feature domination is therefore not purchased with an increased computational burden.

## 4. Discussion

The findings of this study support the central hypothesis underlying FDNet: segmentation performance depends not only on the information preserved through encoder–decoder skip connections but also on how effectively this information is utilized during decoding. By repeating the encoder feature maps without introducing additional learnable parameters through the repetition operation, FDNet improved the segmentation accuracy across all three datasets used in this study. The improvements observed were accompanied by a more balanced distribution of false-positive and false-negative errors, with the best-performing FDNet variant achieving the lowest combined per-slice error rate for each dataset (Table 2). Because the repeated feature maps contained identical information, these gains could not be attributed to the introduction of new feature content. Instead, the results suggest that feature repetition modifies the contribution of encoder information to decoder processing, potentially facilitating the recovery of fine anatomical structures that may be insufficiently represented under conventional skip concatenation.

The error profiles of the comparison methods are informative, and the qualitative examples in Figure 3, Figure 4, Figure 5 and Figure 6 illustrate this. SegNet tended to extend the segmentation into neighboring tissues, producing the highest FPPr of the re-implemented baselines on ADNI and IBSR, which is the spreading of upsampled coarse context described in Section 2.2. U-Net spreads less but misses more, with FN exceeding FP on the example slice in Figure 3 (19 vs. 6), indicating that fine extensions and internal folds are not recovered. The Attention U-Net lies at the far end of this axis: its gates effectively suppress the background, yielding the lowest FPPr on the ADNI and IBSR datasets and the highest PPV, as shown in Table 6 and Table 8. However, the same suppression removes the true structure, and the resulting FN counts (44 pixels on the ADNI slice of Figure 4 and 32 on the IBSR slice of Figure 5) leave it with the lowest DSC of any method trained here. Feature domination acts on the same trade-off by the opposite means: instead of gating the context, it weighs details.

Errors concentrate at the outer boundary of the structure and at its head and tail—where partial-volume mixing, morphological folding, and low tissue contrast coincide, and where the through-plane information discarded by slice-wise processing would be most informative. This is also where the differences between methods are largest, consistent with the view that what distinguishes them is how they handle detail near the boundary rather than the body of the structure. The error asymmetry of Section 2.7 sharpens this: the marginal cost of a false negative relative to a false positive grows as the true-positive area shrinks, and that area is smallest on exactly the thin head and tail slices that separate the methods.

Further qualification arises from the subject-grouped re-evaluation described in Section 2.6. Under the original training configuration, FDNetX2 achieved a subject-level test Dice of 0.8771, compared with 0.9321 under the original slice-level evaluation (Table 2). This difference highlights the potential for optimistic performance estimates when slices from the same subject are allowed to appear in both the training and test sets. However, its magnitude cannot be attributed exclusively to subject-level overlap because the two evaluations also differ in terms of dataset composition, partitioning, and aggregation of segmentation scores. Under the subject-grouped protocol, the development-fold-optimized FDNet variants achieved held-out test Dice scores of 0.9044–0.9050 (Table 3), demonstrating that competitive performance was retained when the subjects were separated across the training, validation, and test partitions. Importantly, the original slice-level protocol was applied consistently to FDNet and the three re-implemented baselines, and the relative ranking observed in Table 2 was preserved under the subject-grouped evaluation. All three FDNet variants significantly outperformed U-Net, Attention U-Net, and SegNet on the held-out ADNI subjects (Holm-adjusted *p* < 0.0001; Table 4). Although FDNet also achieved slightly higher Dice scores than the nnU-Net five-fold ensemble, these differences were not statistically significant after the Holm correction. Thus, the subject-grouped results support the advantage of FDNet over the re-implemented baselines while establishing a more conservative interpretation of its performance relative to nnU-Net. The importance of evaluation protocols is also evident in the literature; some studies reporting particularly high Dice scores on ADNI-derived data do not clearly document subject-level separation (e.g., [62]). Furthermore, Bhosale and Dixit [63] reported a lower performance for Attention U-Net than for plain U-Net, which is consistent with the relative ordering observed in our study.

Dataset differences also shape the absolute error rates. The Decathlon volumes are cropped tightly around the structure and resampled to 32 × 32 × 32, so the foreground occupies approximately 13% of each slice against roughly half a percent in ADNI and IBSR (Table 1). Therefore, per-slice error counts on Decathlon are an order of magnitude larger than those on the other two datasets for every method, and FNPr and FPPr are comparable across methods within a dataset but not across datasets; DSC, normalized by region size, remains comparable in both directions.

A recent ADNI comparison was provided by CSDA-UNet [59], which reported a DSC of 0.9512 against 0.9339 for the FDNetX3. The absolute values should be read cautiously because the two studies used different preprocessing and evaluation protocols, and preprocessing is not a minor factor: in the ablation reported by Wei et al. [59], removing contrast-limited adaptive histogram equalization and Gaussian denoising costs their U-Net 0.2177 DSC (0.9365 → 0.7188) and CSDA-UNet 0.0692 DSC (0.9512 → 0.8820)—the intensity pipeline moves accuracy five to fifteen times as much as the attention modules, so no cross-paper comparison of the two architectures can separate the two effects. Each mechanism can instead be compared with the plain U-Net trained by the same authors on the same data, where the two are close and the ordering reverses: the two cascaded attention modules raise DSC by 0.0147 (0.9365→0.9512), while feature domination raises it by 0.0166 (0.9173 → 0.9339). Feature domination thus recovers a slightly larger share of the gap left by the plain architecture, with 578,530 parameters against 7.76 M—a factor of 13—and no trainable weight.

On Decathlon, the comparison is more straightforward but requires a note on the protocol. The Decathlon hippocampus task comprises 394 volumes split by the organizers into 263 for training and 131 for testing, with the test labels withheld [67]; therefore, scores on that split cannot be computed externally. Wei et al. (2025) reported training and validation figures (0.9907 and 0.8963) rather than a held-out test score [59]; their validation figure is the only one comparable in kind with the 0.9470 obtained here on an unaugmented held-out slice-level split of the labelled data, a difference of 0.0507 in favor of FDNetX3. The same reasoning applies to their reported accuracy (0.9952/0.9832). FDNet is also evaluated on the IBSR, for which no comparably recent result exists.

The Attention U-Net [23] and Shi et al. (2021) hierarchical attention network address the same imbalance by learning where to attend, at the cost of additional parameters and computation [11]. Here, both were outperformed by every FDNet variant on every dataset, and the error decomposition suggests that the reason lies in the direction of the correction rather than its magnitude: the learned gating suppressed the background and true structure together, whereas repetition emphasized detail without removing context. This does not imply that attention is a weaker principle in general; both attention baselines were trained at their published configurations on datasets that were substantially smaller than those in which attention is usually advantageous. However, this suggests that when data are limited, cheaper corrections are competitive. A learnable weighting of the copies (Equation (1) with w_i_ optimized jointly) would interpolate between the two, and is a natural next step.

## 5. Limitations

The following limitations bound the conclusions that can be drawn from this study:

- Segmentation was performed slice-wise on sagittal slices, following the protocol of the studies being compared. Therefore, the network had no through-plane context, and all metrics were per-slice. Volumetric DSC, volume error, and interslice consistency were not evaluated. A volumetric extension of the operator is the most immediate direction for future research.
- All three datasets were pre-cropped using a fixed spatial region that was consistently applied to all volumes within each dataset before slicing (Section 2.1). This procedure did not involve hippocampal localization or subject-specific region of interest detection. Consequently, none of the methods, including the baselines, were required to locate the hippocampus within a complete slice or volume. Therefore, the reported Dice scores should not be interpreted as evidence of performance on the more challenging task of joint localization and segmentation. Evaluation of full slices or volumes, where the network must both identify and segment the hippocampus, is required to determine whether the relative performance of the methods persists without predefined spatial cropping.
- Pairwise differences among the models were assessed using subject-level Wilcoxon signed-rank tests and bootstrap confidence intervals (Section 2.8), with the Holm correction applied within the ADNI comparison; however, the number of held-out subjects (19 in the ADNI) limited their power. This applies with the most force to the three FDNet variants, whose per-slice distributions overlap substantially (Figure 7, Figure 8 and Figure 9) and whose DSC values differ by less than 0.003 on each dataset; the ordering among them should be read as indicative. Statistical significance testing and bootstrap confidence intervals were restricted to the ADNI subject group evaluation; corresponding subject-level statistical comparisons were not performed for Decathlon or IBSR.
- All three datasets are curated research collections; no external or clinical routine data were used, and neither cross-dataset generalization nor robustness to scanner, sequence, or population differences were assessed.
- Dice scores and mean per-slice false-positive and false-negative pixel counts characterize segmentation overlap and error composition but do not establish the accuracy or agreement of hippocampal volume estimates derived from the predicted masks. Such agreement requires additional volumetric analyses, including volume estimation errors and Bland–Altman analysis of predicted versus reference volumes. Because hippocampal volumetry is relevant to the assessment of atrophy in Alzheimer’s disease, the absence of these analyses limits the clinical interpretation of the reported segmentation improvements. Accordingly, the observed gains in spatial overlap should not be interpreted as demonstrated clinical benefits for Alzheimer’s disease assessment.
- The published results summarized in Table 6, Table 7 and Table 8 were obtained using heterogeneous experimental protocols, including differences in slice orientation, dataset size, preprocessing, and evaluation procedures. Consequently, these cross-study comparisons should be interpreted as contextual rather than controlled assessments of the relative model performance. Similarly, the qualitative examples presented in Figure 3, Figure 4, Figure 5 and Figure 6 illustrate representative error patterns but cannot establish their frequency or prevalence across the complete test data sets.
- An additional limitation concerns the comparability of ADNI hippocampal annotations across studies. Although ADNI provides a common imaging resource, studies may differ in their selected subjects, annotation protocols, and evaluation partitions, limiting direct comparison of reported segmentation results. The EADC-ADNI Harmonized Protocol (HarP) provides a standardized framework for manual hippocampal delineation and has been used in previous studies [54,55]. Instead, the present study employed the hippocampal masks described in Section 2.1.1, with the corresponding data partitions and fold definitions documented to support reproducibility. Future evaluation using HarP-compliant annotations and a harmonized subject-level protocol would facilitate more rigorous comparisons with existing methods.

## 6. Conclusions

This study introduced feature domination, a skip-connection modification for U-Net-based hippocampus segmentation, in which identical copies of encoder feature maps are concatenated with their corresponding decoder features. Instead of introducing additional attention or gating modules, FDNet modifies the relative contribution of fine-grained encoder information during feature reconstruction. This approach was motivated by the relationship between false-negative and false-positive predictions and their influence on segmentation overlaps, emphasizing the importance of balancing both error types rather than minimizing either one independently.

Theoretical analysis showed that concatenating identical encoder features does not expand the representable function family because the corresponding convolutional kernels can be algebraically combined. Instead, feature repetition changes the parameterization and optimization dynamics of the encoder contribution. Although this introduces additional convolutional weights, the increase remains modest, with 48,384 parameters per additional copy. The resulting FDNet variants contain 530,146–626,914 parameters, substantially fewer than the larger baseline architectures evaluated in this study. The observed differences among repetition factors suggest that an intermediate degree of feature domination may provide a favorable balance between encoder detail and decoder context, although the underlying optimization mechanisms require further investigation.

Under the original slice-level evaluation protocol, FDNet achieved Dice coefficients of 0.9339 on ADNI, 0.9470 on the Medical Segmentation Decathlon, and 0.9477 on IBSR, outperforming the re-implemented U-Net, Attention U-Net, and SegNet baselines across all three datasets. The best-performing FDNet variant on each dataset also achieved the lowest combined per-slice false-positive and false-negative error count. To address potential optimism associated with slice-level data partitioning, an additional subject-grouped evaluation was conducted on ADNI. Under this more rigorous protocol, the three FDNet variants achieved held-out subject-level Dice scores of 0.9044–0.9050 and significantly outperformed the re-implemented baselines after Holm correction (adjusted *p* < 0.0001). Their numerical advantage over the nnU-Net five-fold ensemble (Dice = 0.9019) did not remain statistically significant after multiple-comparison correction. These findings support the effectiveness of feature domination relative to the evaluated conventional baselines, while indicating competitive, rather than conclusively superior, performance compared with nnU-Net.

Overall, FDNet demonstrates that modifying existing skip-connection pathways can provide competitive hippocampus segmentation performance with a relatively small parameter budget. However, the results should be interpreted within the constraints of the evaluated datasets and experimental protocols. In particular, the absence of a parameter-matched control limits the extent to which performance gains can be attributed exclusively to feature repetition, whereas the use of preprocessed hippocampal regions and the lack of independent cohort validation restrict conclusions regarding broader generalizability and clinical applicability. Further evaluation using parameter-matched comparisons, volumetric segmentation, boundary-based metrics, and independent multi-scanner datasets would help establish the general applicability of the proposed mechanism.

## Figures and Tables

**Table 1:** Characteristics of the three datasets after preprocessing. The foreground ratio is the proportion of the total slice area occupied by the hippocampal label, given before and after augmentation.

Property	ADNI	Decathlon	IBSR
Volumes available	322 (181 participants)	394 (260 labelled)	18
Volumes used	161	260	18
Native volume size (voxels)	166 × 256 × 256	variable	128 × 256 × 256
Patch/resampled size (voxels)	96 × 96 × 96 (centre crop)	32 × 32 × 32 (resampled)	96 × 96 × 120 (centre crop)
Slice plane/size (pixels)	sagittal, 96 × 96	sagittal, 32 × 32	sagittal, 96 × 96
Slices before augmentation	15,456	2080	2160
Slices after augmentation	20,751	4160	3241
Foreground ratio (before → after)	0.38% → 0.62%	12.75% → 13.52%	0.51% → 0.71%

Test slices were held out before augmentation and contained only original slices.

**Table 2:** DSC and mean per-slice false-positive and false-negative pixel counts for the proposed FDNet variants and re-implemented baseline methods on the three datasets. FPPr and FNPr are the mean number of erroneous pixels per slice (Equations (5) and (6)), where a lower value is better. Dec. = Medical Segmentation Decathlon. The best DSC on each dataset is shown in bold and underlined.

Method	ADNI			Decathlon			IBSR		
	DSC	FPPr	FNPr	DSC	FPPr	FNPr	DSC	FPPr	FNPr
FDNetX2	0.9321	3.4273	1.4590	0.9453	12.0327	2.8391	**0.9477**	3.5782	1.3866
FDNetX3	**0.9339**	3.0730	1.6508	**0.9470**	11.3318	3.0255	0.9471	3.4916	1.5227
FDNetX4	0.9327	3.4139	1.4313	0.9446	12.0036	3.0564	0.9453	3.5151	1.6647
SegNet [24]	0.9240	4.0095	1.5045	0.9394	13.1582	3.3718	0.9464	4.8311	0.3807
U-Net [21]	0.9173	4.1931	1.8003	0.9415	12.4455	3.4573	0.9236	5.7605	1.6588
Attention U-Net [23]	0.8910	2.7284	4.6489	0.9175	11.2639	8.9353	0.8459	0.4992	12.0235

**Table 3:** Subject-grouped re-evaluation of ADNI. “Val DSC” is the mean over five subject-grouped development folds; “Test DSC” and the test mIoU/Recall/PPV are on the fixed held-out set of 19 subjects (34 volumes), evaluated once after model selection. FDNet test scores are the mean of the five per-fold models applied to the held-out subjects. The nnU-Net used its five-fold ensemble.

Method	Val DSC	Test DSC	Test mIoU	Test Recall	Test PPV
FDNetX2	0.9069	0.9047	0.9128	0.9123	0.9020
FDNetX3	0.9070	0.9050	0.9130	0.9134	0.9015
FDNetX4	0.9069	0.9044	0.9126	0.9120	0.9015
nnU-Net 2D [36]	0.8999	0.9019	0.9105	0.8939	0.9147
U-Net [21]	0.8919	0.8866	0.8980	0.8948	0.8854
Attention U-Net [23]	0.8900	0.8849	0.8967	0.8924	0.8845
SegNet [24]	0.8430	0.8243	0.8577	0.8108	0.8818

**Table 4:** Statistical comparison on the held-out test set (19 subjects). Per-subject Dice was paired by subject (FDNet and baseline scores averaged over the five-fold models; nnU-Net from its five-fold ensemble). *p*-values are two-sided Wilcoxon signed-rank tests; the final column applies Holm–Bonferroni correction across all nine comparisons. The following values represent 95% bootstrap CIs (10,000 resamples) for each model’s mean test Dice: FDNetX3 [0.898, 0.911], FDNetX2 [0.898, 0.911], FDNetX4 [0.898, 0.911], nnU-Net [0.895, 0.908], U-Net [0.878, 0.895], Attention U-Net [0.875, 0.894], SegNet [0.774, 0.864].

Comparison	Δ Mean DSC	*p* (Wilcoxon)	Holm-Significant
FDNetX3 vs. U-Net [21]	+0.018	<0.0001	Yes
FDNetX3 vs. Attention U-Net [23]	+0.020	<0.0001	Yes
FDNetX3 vs. SegNet [24]	+0.081	<0.0001	Yes
FDNetX3 vs. nnU-Net [36]	+0.0030	0.029	No
FDNetX2 vs. nnU-Net [36]	+0.0028	0.023	No
FDNetX4 vs. nnU-Net [36]	+0.0025	0.026	No
FDNetX3 vs. FDNetX2	+0.0003	0.57	No
FDNetX3 vs. FDNetX4	+0.0005	0.14	No
FDNetX2 vs. FDNetX4	+0.0003	0.52	No

**Table 5:** Width-matched efficiency under the subject-grouped setting. Conv-GFLOPs (Conv2D and Conv2DTranspose only; 1 MAC = 2 FLOPs) are hardware-independent, with peak GPU memory being the TensorFlow allocator peak during training.

Method	conv-GFLOPs	Peak Train. Memory (MiB)
FDNetX2	0.78	2060
FDNetX3	0.91	1755
FDNetX4	1.03	2012
U-Net [21]	0.85	1408
Attention U-Net [23]	0.89	1387
SegNet [24]	11.93	5012

**Table 6:** Performance on the ADNI dataset compared with published results. Rows without a citation to another work were computed in this study. Dashes indicate metrics not reported in the source. Only studies that evaluate 2D slices are listed, so that the DSC values are computed over the same kind of unit as ours; recent 3D volumetric methods are discussed in Section Related Work but are not tabulated because a volume-wise DSC is not comparable to a slice-wise one. Values marked § are taken from Table 6, Table 7 and Table 8 of Wei et al. (2025) [59] and include the plain U-Net that was trained under their own pipeline, which is the reference point for their reported gain. Two of their metrics were omitted rather than transferred because the definitions differed: their IoU was the foreground Jaccard index and not the class-averaged mIoU used here (they reported 0.9345 on ADNI), and their accuracy was computed on zero-padded 256 × 256 axial slices rather than 96 × 96 sagittal ones, so it reflected a different background fraction. Values shown in bold are the largest values in each column.

Method	DSC	mIoU	Recall	Accuracy	PPV	FNPr + FPPr
Chen et al. (2017) [30], multi-view Ensemble-Net	0.8947	-	-	-	-	-
Thyreau et al. (2018) [43]	0.8500	-	-	-	-	-
Cao et al. (2018) [31], multi-task network	0.8930	-	-	-	-	-
Hou et al. (2019) [32]	0.9190	-	-	-	0.9260	-
Folle et al. (2019) [33]	0.8820	-	-	-	-	-
Ataloglou et al. (2019) [46]	0.9015	-	-	-	-	-
Brusini et al. (2020) [34], shape-informed U-Net	0.8556	-	0.8339	-	0.8803	-
Liu et al. (2020) [44]	0.8700	-	0.8970	-	0.8460	-
Shi et al. (2021) [11]	0.9124	-	-	-	-	-
Yousefi-Banaem & Malekzadeh (2021) [37]	0.9230	0.9293	0.9650	-	0.9040	-
Yue et al. (2023) [45], 2D	0.7809	-	-	-	-	-
Yue et al. (2023) [45], 3D	0.8186	-	-	-	-	-
Huang et al. (2023) [38], feedback chain network	0.9236	0.9285	0.9261	0.9350	-	-
U-Net baseline trained by Wei et al. (2025) [59] * (§)	0.9365	-	0.9587	-	0.9439	-
Wei et al. (2025) [59], CSDA-UNet ** (§)	**0.9512**	-	0.9553	0.98	0.9644	-
U-Net [21]	0.9173	0.9233	0.9486	0.9994	0.8886	5.99
Attention U-Net [23]	0.8910	0.9015	0.8672	0.9992	**0.9217**	7.38
SegNet [24]	0.9240	0.9290	0.9570	0.9994	0.8933	5.51
Shi’s method [11], own augmentation	0.9025	0.9097	0.9041	0.9972	0.9010	25.87
nnU-Net [36]	0.9332	0.9371	0.9549	0.9995	0.9124	4.79
FDNetX2 (ours)	0.9321	0.9362	0.9583	0.9995	0.9074	4.89
FDNetX3 (ours)	0.9339	**0.9377**	0.9529	0.9995	0.9156	**4.72**
FDNetX4 (ours)	0.9327	0.9367	**0.9591**	0.9995	0.9077	4.85

* Wei et al. [59] reported train and validation scores rather than a held-out test score; the validation value is quoted here, and their train value of 0.9907 is not comparable with the test scores in this table. ** As in Table 6, the accuracy given for that row is the value reported by those authors for the same validation split (their training figure is 0.9952), and their IoU of 0.8132 is a foreground Jaccard index rather than a class-averaged mIoU and is therefore not entered.

**Table 7:** Performance on the medical segmentation Decathlon dataset compared with published results. The conventions are presented in Table 6. Values shown in bold are the largest values in each column.

Method	DSC	mIoU	Recall	Accuracy	PPV	FNPr + FPPr
Folle et al. (2019) [33]	0.8820	-	-	-	-	-
Ataloglou et al. (2019) [46]	0.8835	-	-	-	-	-
Shi et al. (2021) [11]	0.9038	-	-	-	-	-
Ranem et al. (2022) [35]	0.8976	-	-	-	-	-
Huang et al. (2023) [38]	0.9311	0.9001	0.8950	0.9382	-	-
CSDA-UNet [59]	0.8963	-	-	0.9832	-	-
U-Net [21]	0.9415	0.9358	0.9737	0.9845	0.9114	15.90
Attention U-Net [23]	0.9175	0.9118	0.9264	0.9787	0.9094	20.20
SegNet [24]	0.9394	0.9336	0.9743	0.9838	0.9069	16.53
Shi’s method [11], own augmentation	0.9141	0.9086	0.9113	0.9762	**0.9204**	24.34
FDNetX2 (ours)	0.9453	0.9399	**0.9784**	0.9855	0.9144	14.87
FDNetX3 (ours)	**0.9470**	**0.9417**	0.9770	**0.9860**	0.9189	**14.36**
FDNetX4 (ours)	0.9446	0.9391	0.9767	0.9853	0.9145	15.06

**Table 8:** Performance on the IBSR dataset compared with the published results. The conventions are listed in Table 6. Values shown in bold are the largest values in each column.

Method	DSC	mIoU	Recall	Accuracy	PPV	FNPr + FPPr
Rousseau et al. (2011) [41]	0.8300	-	-	-	-	-
Zarpalas et al. (2014) [42]	0.8520	-	-	-	-	-
Huang et al. (2023) [38]	0.9193	0.8987	0.9011	0.9380	-	-
U-Net [21]	0.9236	0.9287	0.9643	0.9992	0.8870	7.42
Attention U-Net [23]	0.8459	0.8658	0.7410	0.9987	0.9859	12.52
SegNet [24]	0.9464	0.9489	**0.9918**	0.9994	0.9050	5.21
Shi’s method [11]	0.9011	0.9078	0.8934	0.9957	0.9090	17.52
FDNetX2 (ours)	**0.9477**	**0.9501**	0.9701	**0.9995**	0.9264	**4.96**
FDNetX3 (ours)	0.9471	0.9495	0.9672	0.9994	**0.9280**	5.01
FDNetX4 (ours)	0.9453	0.9479	0.9641	**0.9995**	0.9272	5.18

**Table 9:** Recently published results on the same datasets are reported here for completeness but are not included in Table 6, Table 7 and Table 8 for conciseness. The DSC values were those reported by the cited authors and were not recalculated.

Study	Dataset	Model	DSC	Accuracy	Evaluation Unit	Why Not Directly Comparable
Das & Kalita (2022) [68]	ADNI	Modified 3D U-Net	0.9343	0.90	Volume	Volumetric score; the study targets disease-stage classification
Nisha et al. (2022) [69]	ADNI	Deep integration model	0.85	0.95	Not stated	The evaluation unit was not stated in the accessible source.
Lei et al. (2023) [70]	Decathlon	Morphological ViT	0.886–0.900	-	Substructure	Segments hippocampal substructures, so DSC is per substructure
Xiao et al. (2024) [28]	Decathlon	Swin-UNETR	0.878	-	Volume	A comparison baseline within another study, not that study’s proposed method
Xiao et al. (2024) [28]	Three hippocampal sets	Light3DHS	not reproduced here	-	Volume	Volumetric score; datasets and splits differ
Ding et al. (2024) [61]	ADNI/Decathlon	PCFR-Net	0.9386/0.9274	-	Not stated	Evaluation unit not stated in the accessible record
Khan et al. (2025) [54]	EADC-ADNI HarP	Resource-efficient 3D U-Net	0.8838	-	Volume	Volumetric score on a different dataset
Pusparani et al. (2026) [60]	ADNI	U-Net [21]	0.784–0.890	-	Slice, stratified	Reported separately by diagnostic group and hemisphere rather than pooled

**Table 10:** Computational efficiency of the evaluated methods on the ADNI dataset. The parameter count, stored model size, mean training time per epoch, and inference time per input slice were reported. Lower values indicate lower computational cost.

Method	Total Parameters	Weight Size	Relative Parameter Count (FDNetX2 = 1.0×)	Training Time/Epoch (s)	Inference Time/Slice (ms)
U-Net [21]	34,512,258	134,902 KB	65.1×	50.98	2.11
Attention U-Net [23]	37,306,190	145,942 KB	70.4×	66.59	2.50
SegNet [24]	88,092,546	344,330 KB	166.2×	79.28	3.03
Shi’s method [11]	3,837,697	15,563 KB	7.2×	18.36 *	3.67 *
CSDA-UNet [59]	7,760,000	not reported	14.6×	NR **	NR **
nnU-Net [36]	7,766,344	30,338 KB	14.6×	21.9	1.98
FDNetX2 (ours)	530,146	2140 KB	1.0×	8.90	0.70
FDNetX3 (ours)	578,530	2329 KB	1.1×	9.73	0.75
FDNetX4 (ours)	626,914	2518 KB	1.2×	10.83	0.99

* Shi’s method forms each input from three consecutive slices stacked as three channels; therefore, its sample counts and timing values are not directly comparable with those of the single-slice models. ** NR: Not Reported. CSDA-UNet was not re-implemented in the present study. Wei et al. [59] reported a total ADNI training time of 3 h 45 min and an inference time of approximately 0.38 s per slice. Because the number of training epochs used for the ADNI experiment was not specified in the reported computational analysis, training time per epoch could not be derived directly.

## Data Availability

The three datasets analysed in this study are available online: ADNI from the LONI Image Data Archive (adni.loni.usc.edu, available upon registration and subject to the ADNI data use agreement, accessed on 13 November 2025); the hippocampus task of the Medical Segmentation Decathlon from https://medicaldecathlon.com (accessed on 2 December 2025); and IBSR from nitrc.org (accessed on 8 December 2025). The source code, trained model weights, and the split definitions used for training, validation, and testing are available at https://github.com/bingolo/FDNet (accessed on 27 October 2026).

## Abbreviations

The following abbreviations are used in this manuscript.

AD	Alzheimer’s disease
ADNI	Alzheimer’s Disease Neuroimaging Initiative
BCE	binary cross-entropy
CNN	convolutional neural network
DSC	Dice similarity coefficient
FDNet	feature-dominated U-Net
FN	false negative
FNPr	rate of false-negative prediction per slice
FP	false positive
FPPr	rate of false-positive prediction per slice
IBSR	Internet Brain Segmentation Repository
IoU	intersection over union
MCI	mild cognitive impairment
mIoU	mean intersection over union
MICCAI	Medical Image Computing and Computer Assisted Intervention
MRI	magnetic resonance imaging
NITRC	Neuroimaging Tools and Resources Collaboratory
PPV	positive predictive value
ReLU	rectified linear unit
TN	true negative
TP	true positive
